# The Cardiac Syndecan-2 Interactome

**DOI:** 10.3389/fcell.2020.00792

**Published:** 2020-08-28

**Authors:** Sabrina Bech Mathiesen, Marianne Lunde, Maria Stensland, Marita Martinsen, Tuula A. Nyman, Geir Christensen, Cathrine Rein Carlson

**Affiliations:** ^1^Institute for Experimental Medical Research and Oslo University Hospital, University of Oslo, Oslo, Norway; ^2^Department of Immunology, Institute of Clinical Medicine, University of Oslo, Oslo, Norway; ^3^K.G. Jebsen Center for Cardiac Research, University of Oslo, Oslo, Norway

**Keywords:** syndecan-2, syndecan, proteoglycans, interactome, cardiac, heart, CAVIN, junctophilin

## Abstract

The extracellular matrix (ECM) is important in cardiac remodeling and syndecans have gained increased interest in this process due to their ability to convert changes in the ECM to cell signaling. In particular, syndecan-4 has been shown to be important for cardiac remodeling, whereas the role of its close relative syndecan-2 is largely unknown in the heart. To get more insight into the role of syndecan-2, we here sought to identify interaction partners of syndecan-2 in rat left ventricle. By using three different affinity purification methods combined with mass spectrometry (MS) analysis, we identified 30 novel partners and 9 partners previously described in the literature, which together make up the first cardiac syndecan-2 interactome. Eleven of the novel partners were also verified in HEK293 cells (i.e., AP2A2, CAVIN2, DDX19A, EIF4E, JPH2, MYL12A, NSF, PFDN2, PSMC5, PSMD11, and RRAD). The cardiac syndecan-2 interactome partners formed connections to each other and grouped into clusters mainly involved in cytoskeletal remodeling and protein metabolism, but also into a cluster consisting of a family of novel syndecan-2 interaction partners, the CAVINs. MS analyses revealed that although syndecan-2 was significantly enriched in fibroblast fractions, most of its partners were present in both cardiomyocytes and fibroblasts. Finally, a comparison of the cardiac syndecan-2 and -4 interactomes revealed surprisingly few protein partners in common.

## Introduction

To cope with injury and mechanical stress, the heart can change its shape and function, a process associated with alterations of the extracellular matrix (ECM) and progression toward heart failure (Cohn et al., 2000). At the cellular level, this includes hypertrophy or death of cardiomyocytes and activation of fibroblasts to ECM producing myofibroblasts, which manifests itself as hypertrophy of the myocardium and stiffening through fibrosis (Cohn et al., 2000). Proteoglycans are emerging as important players in ECM remodeling in the heart, including members of the syndecan family (Christensen et al., 2019). Syndecan-4 has been shown to be a pro-remodeling molecule, acting in both cardiomyocytes and fibroblasts (Finsen et al., 2011; Herum et al., 2015). Knock-out of syndecan-4 in mice has been shown to hinder development of pressure overload induced hypertrophy and stiffening of the myocardium through the calcineurin-NFAT pathway and collagen crosslinking (Finsen et al., 2011; Herum et al., 2013; Herum et al., 2015). Although syndecan-4 has been identified as an important signaling mediator, little is known about its close relative, syndecan-2 in the heart. Both syndecan-2 and -4 are expressed in the heart and upregulated following aortic banding (Strand et al., 2013).

The vertebrate syndecan family arose as a result of two rounds of gene duplication, resulting in four family members where syndecan-2 and -4 form one subfamily (Chakravarti and Adams, 2006). While syndecan-4 is ubiquitously expressed, syndecan-2 is primarily expressed in cells from mesenchymal origin, including fibroblasts, endothelial and neuronal cells and is upregulated during development (David et al., 1993; Kim et al., 1994; Ethell and Yamaguchi, 1999; Chen E. et al., 2004). However, syndecan-2 expression has also been observed in cardiomyocytes (Balza and Misra, 2006). Syndecan-2 has been implicated in diverse cellular events, including highly dynamic processes such as angiogenesis and cancer metastasis, but also in formation of mature structures like dendritic spines and control of ECM assembly, all of which appear to require the intact cytoplasmic domain (Ethell and Yamaguchi, 1999; Klass et al., 2000; Chen E. et al., 2004; Essner et al., 2006; Lee et al., 2011; Lim and Couchman, 2014). Its cytoplasmic tail is short, can be subdivided into three regions and has no known intrinsic enzymatic activity, but can connect to multiple proteins (Couchman, 2010). The membrane proximal C1 region can connect to ezrin, which associates with the actin cytoskeleton (Granes et al., 2000). The membrane distal C2 region binds PDZ domain proteins and is mainly involved in intracellular trafficking (Ethell et al., 2000; Zimmermann et al., 2005). The C1 and C2 regions are in common, whereas the middle V (variable) region is unique to each of the syndecans and is probably responsible for syndecan specific signaling (Couchman et al., 2015).

To better understand the role of syndecan-2 in the heart, we here aimed to identify cytoplasmic interaction partners of syndecan-2 in rat left ventricle (LV) and to construct the cardiac syndecan-2 interactome.

## Results

### Combining Three Affinity Purification Approaches to Capture Syndecan-2 Interaction Partners

**FIGURE 1:**
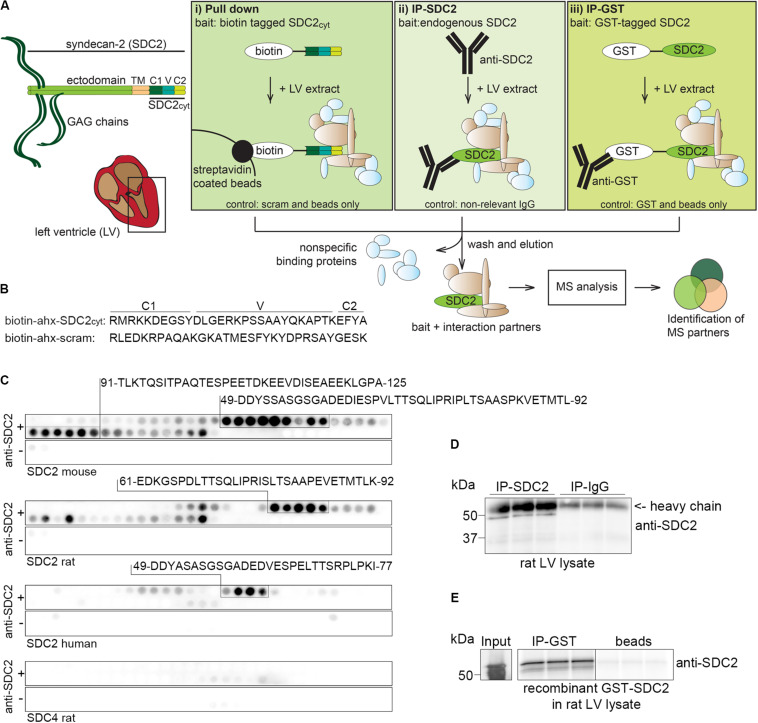
Experimental design to identify syndecan-2 interaction partners in LV lysate. **(A)** Schematic illustration of the three different AP-MS experiments used to capture syndecan-2 interaction partners: (i) pull down with biotinylated peptides covering the cytoplasmic tail of syndecan-2 (SDC2_cyt_, left panel), (ii) precipitation of endogenous cardiac syndecan-2 using anti-SDC2 (middle panel) and (iii) fishing with recombinant GST-tagged syndecan-2 and precipitation by GST antibodies (right panel). After washing away non-specific proteins, putative interaction partners were eluted, trypsin digested and analyzed by mass spectrometry. **(B)** Protein sequences of the SDC2_cyt_ and a scrambled (scram) control peptide used for the pull down approach described in **(Ai)**. An ahx linker was inserted between the biotin-tag and the sequence to avoid steric hindrance. **(C)** Epitope mapping of the syndecan-2 antibody (anti-SDC2) used in **(Aii)**, using peptide array membranes containing 20-mer overlapping syndecan-2 (mouse, rat, and human) and syndecan-4 (rat) peptides. **(D)** Validation of anti-SDC2 in immunoprecipitation experiments of endogenous syndecan-2 in rat LV lysates. **(E)** Validation of the GST antibody in immunoprecipitation experiments of recombinant GST-syndecan-2 in rat LV lysates.

We identified putative syndecan-2 interaction partners from rat LV lysates by combining three AP approaches with MS. Figure 1A depicts the experimental design, emphasizing the three different baits and respective controls (bottom of the boxes). The left panel (i) shows biotinylated peptides of the syndecan-2 cytoplasmic domain (SDC2_cyt_) used as bait to pull down interaction partners by streptavidin coated beads. An ahx linker was inserted in between the biotin-tag and the syndecan-2 cytoplasmic sequence to avoid steric hindrance (Figure 1B, upper sequence). A scrambled syndecan-2 peptide (scram) (Figure 1B, lower sequence) and beads without peptides (beads only) were used as negative controls. The middle panel of Figure 1A, (ii), illustrates IP with anti-syndecan-2 (anti-SDC2) where endogenous syndecan-2 was used as bait. Specificity of the antibody was demonstrated by overlaying anti-SDC2 onto membranes with spot-synthesized 20-mer overlapping peptides, which covered the protein sequence of either mouse, rat or human syndecan-2 or rat syndecan-4. This revealed an ectodomain epitope in syndecan-2 across species, which left the cytoplasmic tail free to interact with protein partners (Figure 1C). Importantly, the antibody only recognized syndecan-2 and showed no cross reactivity toward rat syndecan-4 (Figure 1C, lower panel). Anti-SDC2 was also demonstrated to be able to precipitate endogenous syndecan-2 from rat LV lysates (Figure 1D). The right panel of Figure 1A, (iii), illustrates IP-GST where a recombinant N-terminal GST-tagged full-length syndecan-2 protein (GST-SDC2) was used as bait to capture interaction partners. Recombinant GST without syndecan-2 and beads were used as negative controls. The GST antibody was demonstrated to precipitate GST-SDC2 in rat LV lysates prior to the large AP-MS analysis (Figure 1E). Following the three different APs, the precipitated interaction partners were subjected to trypsin digestion and subsequent MS analysis (Figure 1A, bottom part).

### Identification of 30 Novel Syndecan-2 Interaction Partners

**FIGURE 2:**
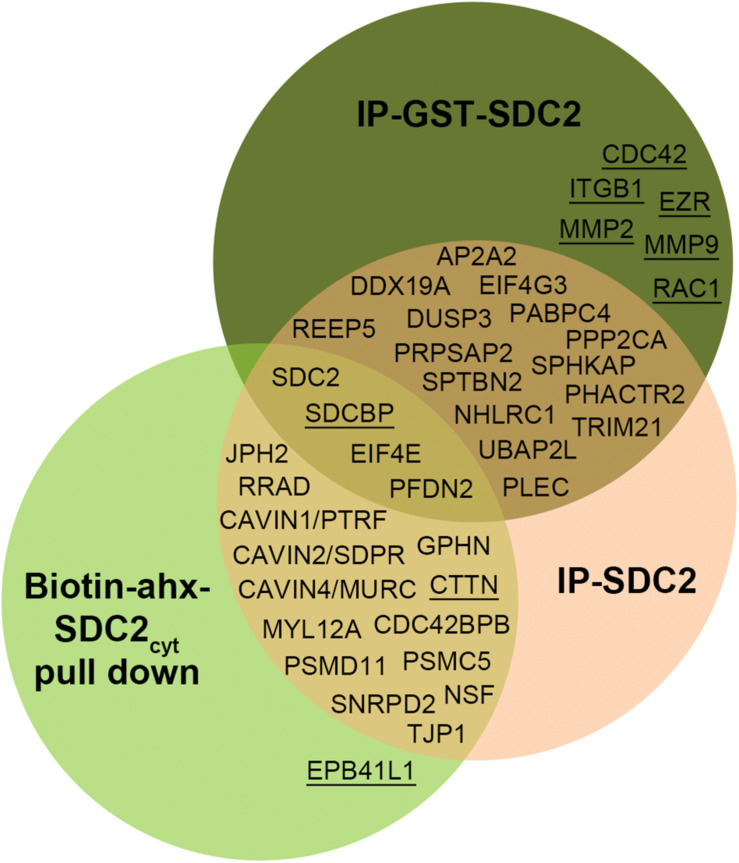
Venn diagram depicting the distribution of syndecan-2 interaction partners in the three AP-MS approaches. Overall, 30 novel putative syndecan-2 partners were identified and they are given in the circles representing the AP-MS methods where they were identified. Syndecan-2 interaction partners previously described in the literature are underlined. The literature partner SDCBP was found in all three AP-MS approaches and the literature partner CTTN was found in two.

All APs were done in biological triplicates. To be considered as a syndecan-2 interaction partner, proteins had to be identified in IP-SDC2 (Figure 2 peach colored circle) and additionally in either IP-GST (Figure 2 dark green circle) or pull down with SDC2_cyt_ (Figure 2 light green circle). Proteins known to be confined in the nucleus, ribosome and mitochondria were excluded since they were regarded as contaminants. Overall, 30 novel syndecan-2 interaction partners were identified by AP-MS with these criteria (hereafter referred to as MS partners) and are listed in Table 1 (detailed overview in Supplementary Table S1). Table 2 summarizes syndecan-2 protein interaction partners described in the literature across tissues and species (hereafter referred to as literature partners). Importantly, both the literature partners cortactin (CTTN) and syntenin-1 (SDCBP) were identified in either two or three of the AP-MS approaches (underlined in Figure 2 and in Tables 1, 2). In addition, seven literature partners were identified by fishing with the SDC2_cyt_ peptide or GST-SDC2 in the LV lysate. These were the cell division control protein 42 homolog (CDC42), band 4.1-like protein 1 (EPB41L1), ezrin (EZR), β1 integrin (ITGB1), matrix metalloproteinase-2 (MMP2), matrix metalloproteinase-9 (MMP9) and ras-related C3 botulinum toxin substrate 1 (RAC1) (underlined in Figure 2 and Table 2, *n* = 3).

**TABLE 1:** Thirty novel syndecan-2 interaction partners identified by AP-MS.

Gene	Protein (Uniprot)	AP-MS (*n* = 3)	Molecular function (HPRD)
*AP2A2^(*a) FB*^*	AP-2 complex subunit alpha-2	IP-SDC2, GST-SDC2	Transporter activity
*CAVIN1/PTRF^*CM*^*	Caveolae-associated protein 1	IP-SDC2, SDC2_cyt_	Transcription regulator activity
*CAVIN2/SDPR^(*a) FB*^*	Caveolae-associated protein 2	IP-SDC2, SDC2_cyt_	Serine-type peptidase activity
*CAVIN4/MURC^*CM*^*	Caveolae-associated protein 4	IP-SDC2, SDC2_cyt_	Unknown
*CDC42BPB/MRCKβ*	Serine/threonine-protein kinase MRCK beta	IP-SDC2, SDC2_cyt_	Protein serine/threonine kinase activity
*CTTN^(*a) FB*^*	Src substrate cortactin	IP-SDC2, SDC2_cyt_	Cytoskeletal protein binding
*DDX19A^(*a)*^*	ATP-dependent RNA helicase DDX19A	IP-SDC2, GST-SDC2	Unknown
*DUSP3*	Dual-specificity phosphatase 3	IP-SDC2, GST-SDC2	Protein tyrosine/serine/threonine phosphatase activity
*EIF4E^(*a)*^*	Eukaryotic translation initiation factor 4E	IP-SDC2, GST-SDC2, SDC2_cyt_	Translation regulator activity
*EIF4G3*	Eukaryotic translation initiation factor 4 gamma, 3	IP-SDC2, GST-SDC2	Translation regulator activity
*GPHN*^*CM*^	Gephyrin	IP-SDC2, SDC2_cyt_	Unknown
*JPH2^(*a) CM*^*	Junctophilin-2	IP-SDC2, SDC2_cyt_	Cell adhesion molecule activity
*NHLRC1^(*b)*^*	E3 ubiquitin-protein ligase NHLRC1	IP-SDC2, GST-SDC2	Ubiquitin-specific protease activity
*MYL12A^(*a) CM*^*	Myosin regulatory light chain 12A	IP-SDC2, SDC2_cyt_	Calcium ion binding
*NSF^(*a)*^*	Vesicle-fusing ATPase	IP-SDC2, SDC2_cyt_	ATPase activity
*PABPC4*^*CM*^	Polyadenylate-binding protein 4	IP-SDC2, GST-SDC2	RNA binding
*PFDN2^(*a)*^*	Prefoldin subunit 2	IP-SDC2, GST-SDC2, SDC2_cyt_	Chaperone activity
*PHACTR2^(*b)*^*	Phosphatase and actin regulator 2	IP-SDC2, GST-SDC2	Phosphatase regulator activity
*PLEC*^*CM*^	Plectin	IP-SDC2, GST-SDC2	Cytoskeletal anchoring activity
*PPP2CA*^*CM*^	Serine/threonine-protein phosphatase 2A catalytic subunit alpha isoform	IP-SDC2, GST-SDC2	Protein serine/threonine phosphatase activity
*PRPSAP2*^*CM*^	Phosphoribosyl pyrophosphate synthase-associated protein 2	IP-SDC2, GST-SDC2	Unknown
*PSMC5^(*a) CM*^*	26S protease regulatory subunit 8	IP-SDC2, SDC2_cyt_	Ubiquitin-specific protease activity
*PSMD11^(*a)*^*	26S proteasome non-ATPase regulatory subunit 11	IP-SDC2, SDC2_cyt_	Ubiquitin-specific protease activity
*REEP5*	Receptor expression-enhancing protein 5	IP-SDC2, GST-SDC2	Unknown
*RRAD^(*a,b)*^*	GTP-binding protein RAD	IP-SDC2, SDC2_cyt_	GTPase activity
*SDCBP^(*a) CM*^*	Syntenin-1	IP-SDC2, GST-SDC2, SDC2_cyt_	Receptor signaling complex scaffold activity
*SNRPD2*	Small nuclear ribonucleoprotein Sm D2	IP-SDC2, SDC2_cyt_	RNA binding
*SPHKAP*	A-kinase anchor protein SPHKAP	IP-SDC2, GST-SDC2	Unknown
*SPTBN2*	Spectrin beta chain, non-erythrocytic 2	IP-SDC2, GST-SDC2	Cytoskeletal protein binding
*TJP1/ZO-1^*CM*^*	Tight junction protein ZO-1	IP-SDC2, SDC2_cyt_	Cell adhesion molecule activity
*TRIM21^(*b)*^*	E3 ubiquitin-protein ligase TRIM21	IP-SDC2, GST-SDC2	Ribonucleoprotein
*UBAP2l*	Ubiquitin-associated protein 2-like	IP-SDC2, GST-SDC2	Unknown

**^(a)^*Precipitated also HA-SDC2 in HEK293 cells. *^(b)^*Not found in rat neonatal cardiomyocytes or fibroblasts by MS analyses. *^*CM*^*Enriched in rat neonatal cardiomyocytes. *^*FB*^*Enriched in rat neonatal fibroblasts. Underlined proteins have previously been described in the literature.*

**TABLE 2:** Protein interaction partners previously reported for syndecan-2 or a conserved syndecan motif across species and tissue^a^.

Gene	Protein	Where in SDC2	Evidence^c^	Biological role	Reference	AP-MS^b^
*ARHGAP35*^CM^	Rho GTPase-activating protein 35/p190ARhoGAP		Functional interaction	Actin cytoskeletal organization (SDC2 might control localization of p190RhoGAP)	Lim and Couchman, 2014	
*CASK*	Peripheral plasma membrane protein CASK	C2 (motif: EFYA) Direct	Peptide binding assays, Y2H and co-localization	Link to actin cytoskeleton and protein 4.1	Cohen et al., 1998; Hsueh et al., 1998	
*CAV2*	Caveolin-2		Co-IP	Regulation of adhesion. SDC2 might also be in complex with CAV1 (Shi et al., 2013)	Huang and Chuang, 2006; Lim et al., 2015	
*CDC42^CM^*	Cell division control protein 42 homolog		Cooperative interaction Cell adhesion studies	Filopodia formation in fibroblasts	Granes et al., 1999	IP-GST
*CDH1*	Cadherin-1/E-cadherin		Functional interaction	SDC2 promote E-cadherin shedding probably by MMP7	Jang et al., 2016	
*CTTN*	Cortactin	C1 (Motif: RMKKKDEGSY) Indirect	Affinity chromatography	Cortical actin organization	Kinnunen et al., 1998; Halden et al., 2004	IP-SDC2, SDC2_cyt_
*IL8/CXCL8*	Interleukin-8	HS chains and possibly ectodomain	Co-IP, isothermal titration	Inflammation signaling	Halden et al., 2004	
*CXCR2*	C-X-C chemokine receptor type 2		Co-IP, co-localization	After stimulation CXCR2 and SDC2 co-localize	Renga et al., 2012	
*DNM2*	Dynamin-2	C1 Indirect	Co-IP, Y2H, pull down		Yoo et al., 2005	
*EPB41L1*	Band 4.1-like protein 1/Protein 4.1N	Cytoplasmic domain	Pull down	Formation of quaternary complex InsP3R1–4.1N–CASK–SDC2 in brain	Maximov et al., 2003	SDC2_cyt_
*EPHB2*	EphB2 receptor tyrosine kinase/Ephrin type-B receptor 2	C1 + V region Direct	Co-IP, phosphorylation assay, co-localization	Phosphorylation on Y179 and Y191 (human numbering) Phosphorylation causes SDC2 clustering and is important for dendritic spine maturation	Ethell et al., 2001	
*EZR^CM^*	Ezrin	C1 (motif: DEGSYD) Direct	Co-IP, pull-down with peptides, co-localization, triton resistant complex	Link to actin cytoskeleton Interaction enhanced by RhoA	Granes et al., 2000; Granés et al., 2003	IP-GST
*FYN*	Fyn	C1	Affinity chromatography	Protein complex in brain	Kinnunen et al., 1998	
**GIPC1**	Synectin/PDZ domain-containing protein GIPC	C2	Y2H, Co-IP	Migration	Gao et al., 2000	
*ITGA2*	Integrin α2		Cooperative interaction, cell adhesion experiments	Cancer migration	Choi et al., 2009	
*ITGA5*	Integrin α5		Cooperative interaction, cell adhesion experiments	Stress fiber formation in lung carcinoma cell line	Kusano et al., 2000	
**ITGAL**	Integrin alpha-L (LFA-1)	Cytoplasmic domain	Cooperative interaction	SDC2 regulates the activity conformation of ITGAL	Rovira-Clave et al., 2014	
*ITGAV*^CM^	Integrin alpha-V		Cooperative interaction	Localization and virus entry	Cheshenko et al., 2007	
*ITGB1*	Integrin β1	Indirect ectodomain	Cooperative interaction, cell adhesion experiments	Adhesion	Kusano et al., 2000; Whiteford et al., 2007	IP-GST
**ITGB4**	Integrin β4	C2	Y2H	Cell spreading	Wang et al., 2010	
*ITPR1*^FB^	Inositol 1,4,5-trisphosphate receptor type 1/InsP3R1	Cyt domain Indirect	Pull down with SDC2 cytoplasmic peptides	Quaternary complex with SDC2-CASK-EPB41L1 in brain	Maximov et al., 2003	
*MMP2^FB^*	Matrix metalloproteinase-2/72 kDa type IV collagenase	Direct?	Ability to shed from cell surface (MMP added to cells, cell lysate dot blotted)	Shedding	Fears et al., 2006	IP-GST
*MMP7*	Matrix metalloproteinase-7	Direct, ectodomain	Co-IP, overlay assay	SDC2 might regulate processing of pro-MMP7	Ryu et al., 2009	
*MMP9*	Matrix metalloproteinase-9	Direct?	Ability to shed from cell surface	Shedding	Fears et al., 2006	IP-GST
*MMP14*^FB^	Matrix metalloproteinase-14	Direct	Cleavage analysis	Shedding of SDC2 by membrane anchored MMP14	Lee et al., 2017	
*NF1*	Neurofibromin	Membrane proximal region of cytoplasmic domain Direct	Y2H, pull down, co-localization	Syndecans might localize neurofibromin to the membrane	Hsueh et al., 2001	
*NOTCH3*	Neurogenic locus notch homolog protein 3		Co-IP	SDC2 might amplify notch signaling	Zhao et al., 2012	
*PRKC –PRKCA –PRKCB –PRKCG*	Protein kinase C	V region Direct	*In vitro* peptide phosphorylation studies, PKC inhibitor blocks phosphorylation	Phosphorylation on S187 and S188 (human numbering)	Prasthofer et al., 1995; Oh et al., 1997	
**PTPRJ**	Protein tyrosine phosphatase receptor CD148/Receptor-type tyrosine-protein phosphatase eta	Membrane proximal region of shed SDC2 Direct	Solid-phase binding assay	Adhesion	Whiteford et al., 2011	
**RAC1**	Ras-related C3 botulinum toxin substrate 1		Functional interaction, overexpression of SDC2 increase Rac1 activity in cancer cells	Cancer migration	Choi et al., 2010	IP-GST
*RACK1/NB2L1*	Receptor of activated protein C kinase 1	FL	Co-IP, affinity chromatography	Scaffolding and downstream signaling	Huang et al., 2005a, b; Renga et al., 2012	
*RASA1*	Ras GTPase-activating protein 1/P120-GAP	FL	Co-IP	Downstream signaling	Huang et al., 2005a	
**SARM1**	Sarm1/Sterile alpha and TIR motif-containing protein 1	Cytoplasmic domain Direct	Pull down, co-IP	Regulation of dendritic outgrowth	Chen et al., 2011	
*SDC2*^FB^	Syndecan-2	TM: GXXXG	Mutation studies	Oligomerization	Choi et al., 2005; Dews and Mackenzie, 2007	IP-SDC2, IP-GST, SDC2_cyt_
**SDC4**	Syndecan-4	TM: GXXXG		Hetero-oligomerization	Choi et al., 2015	
**SDCBP**	Syntenin/syntenin-1	C2 (motif: EFYA) Direct	Y2H, surface plasmon resonance, peptide binding assays and co-localization	Adaptor and intracellular trafficking	Grootjans et al., 1997	IP-SDC2, IP-GST, SDC2_cyt_
*SRC*	Proto-oncogene tyrosine-protein kinase Src	C1	Affinity purification	Protein complex in brain	Kinnunen et al., 1998	
*TGFBR1*	TGF-beta receptor type-1 (TβRI)		Co-IP, functional studies	SDC2 may attenuate TGF-β1 signaling by internalizing	Shi et al., 2013	
*TGFBR3*	Transforming growth factor beta receptor type 3 (Betaglycan)	Cytoplasmic domain is needed	Co-IP	May be involved in fibrosis	Chen L. et al., 2004	
*TIAM1*	Tiam1	C2 (EFYA)	Co-IP, fluorescence- and NMR-based binding assays	Cell migration	Shepherd et al., 2010	
*TRAPPC4/SBDN*	Trafficking protein particle complex subunit 4/Synbindin	C2 (motif: EFYA) Direct	Co-IP, pull-down, ligand overlay, Y2H and co-localization	Vesicle trafficking (neurons, spine maturation)	Ethell et al., 2000	

**^*a*^*Core-protein binders only, partners who bind exclusively to glycosaminoglycan (GAG) chains were excluded. *^*b*^*This includes all three affinity purification methods (each in triplicates, p < 0.05). Pull down with SDC2 peptides is given as “SDC2_*cyt*_”. Expanded information is given in Supplementary Table S1. *^*c*^*Functional and cooperative interactions refer to partners where there is no evidence of direct binding, but good evidence that the two molecules collaborate together. Previously scientists have referred to the syndecan-integrin interactions as cooperation and we have kept that nomenclature here. ns, not significant. *^*CM*^*Enriched in rat neonatal cardiomyocytes. *^*FB*^*Enriched in rat neonatal fibroblasts.*

### Verification of 11 Syndecan-2 Interaction Partners in HEK293 Cells

**FIGURE 3:**
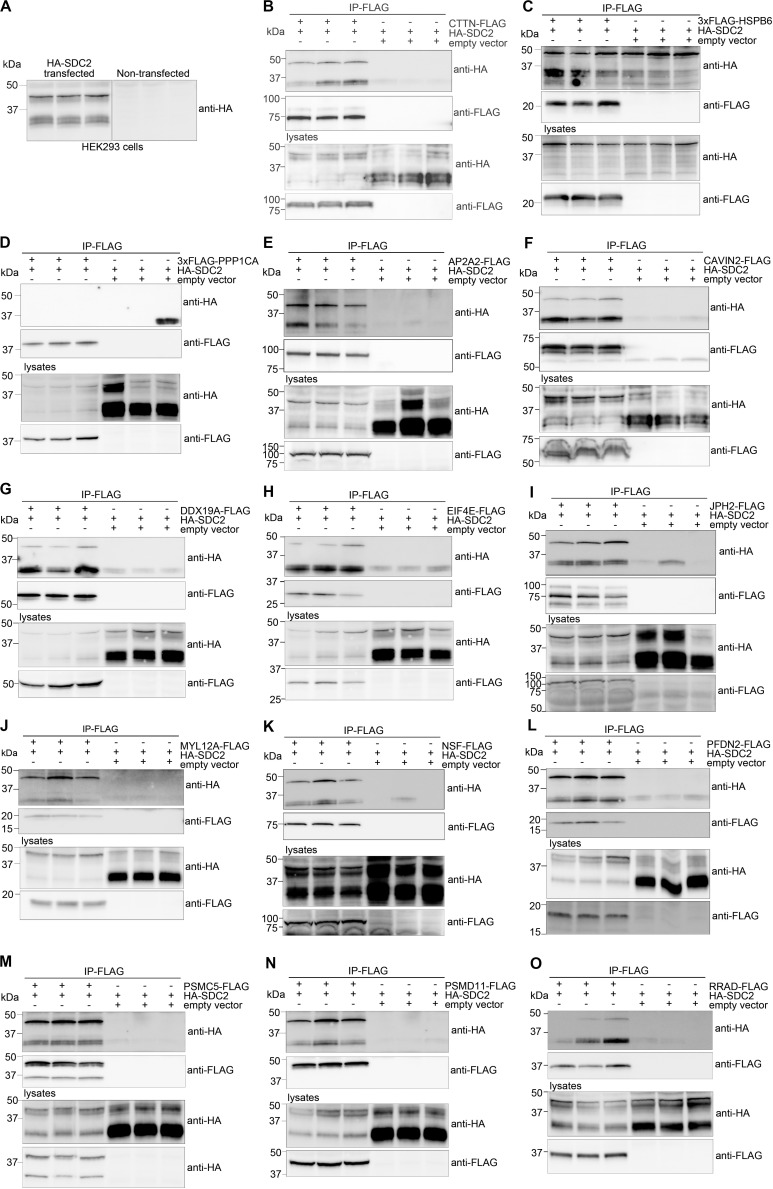
Experimental design for validation of MS partners in HEK293 cells. The MS partner was FLAG-tagged and expressed together with HA-tagged syndecan-2 (HA-SDC2) in HEK293 cells and subjected to IP-FLAG (reversed set-up of Figure 1A). **(A)** Lysates from non-transfected or HA-SDC2 transfected HEK293 cells were developed with anti-HA. IP-FLAG in lysate from HEK293 cells co-transfected with HA-SDC2 and **(B)** CTTN-FLAG (pos. ctrl), **(C)** 3xFLAG-HSPB6 (neg. ctrl.), **(D)** 3xFLAG-PPP1CA (neg. ctrl.), **(E)** AP2A2-FLAG, **(F)** CAVIN2-FLAG, **(G)** DDX19A-FLAG, **(H)** EIF4E-FLAG, **(I)** JPH2-FLAG, **(J)** MYL12A-FLAG, **(K)** NSF-FLAG, **(L)** PFDN2-FLAG, **(M)** PSMC5-FLAG, **(N)** PSMD11-FLAG and **(O)** RRAD-FLAG or an empty vector (neg. ctrl.). The IPs (two upper membranes) and lysates (two lower membranes) were analyzed with anti-HA and anti-FLAG. The HA-SDC2 blots are cropped to show the core protein.

We decided to verify selected MS partners in additional binding studies using HEK293 cells and chose a set-up that reversed the strategy used for MS. Two syndecan-2 bands of approximately 30 (doublet) and 45 kDa were detected when HA-tagged syndecan-2 (HA-SDC2) was analyzed by immunoblotting (Figure 3A). The core domain of human syndecan-2 is predicted to approximately 23 kDa and is known to form SDS resistant dimers through the transmembrane domain. Eleven of the 30 MS partners were expressed with a FLAG-tag together with HA-SDC2 in HEK293 cells and subjected to IP-FLAG. Cortactin (CTTN) was included as a positive control and heat shock protein beta-6 (HSPB6) and serine/threonine-protein phosphatase PP1-alpha catalytic subunit (PPP1CA) were included as negative controls. The latter controls were included to ensure that protein precipitation was not due to simple overexpression. As expected, CTTN precipitated HA-SDC2 (Figure 3B), whereas the negative controls did not show specific binding (Figures 3C,D). The 11 MS partners that precipitated HA-SDC2 were AP-2 complex subunit alpha-2 (AP2A2), caveolae-associated protein 2 (CAVIN2), ATP-dependent RNA helicase DDX19A (DDX19A), eukaryotic translation initiation factor 4E (EIF4E), junctophilin-2 (JPH2), myosin regulatory light chain 12A (MYL12A), vesicle-fusing ATPase (NSF), prefoldin subunit 2 (PFDN2), 26S protease regulatory subunit 8 (PSMC5), 26S proteasome non-ATPase regulatory subunit 11 (PSMD11) and GTP-binding protein RAD (RRAD) (Figures 3E–O, respectively).

### Overview of the Syndecan-2 Interactome

**FIGURE 4:**
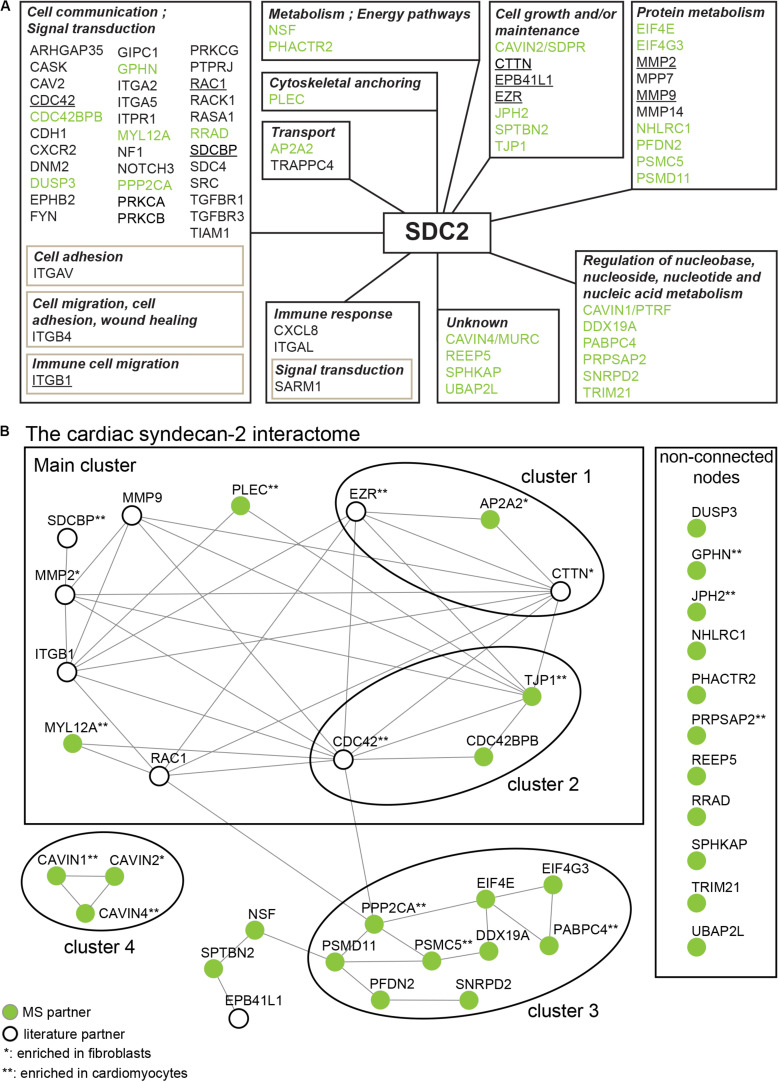
The syndecan-2 interactome. **(A)** The 30 novel syndecan-2 interaction partners identified in this study (green), together with the 41 syndecan-2 partners previously described in the literature (Table 2), were grouped according to the GO annotation biological process (hprd.org). Partners in brown boxes represent subcategories. Syndecan-2 literature partners also found in this study are underlined. **(B)** The 30 novel syndecan-2 partners (green circles) and the 9 literature partners that were also found in this study (white circles) constitute the cardiac syndecan-2 interactome. The proteins were assessed for interconnectivity via STRING database network analysis (Szklarczyk et al., 2017). The literature partners CDC42, ITGB1, EZR, MMP2, MMP9, RAC1, and EPB41L1 were identified in only one of our AP-MS approaches (Figure 2), but were included since they connected well to the novel MS partners and might thus play a role with syndecan-2 in heart. The literature partners CTTN and SDCBP were identified in at least two MS approaches (Figure 2).

To get a more comprehensive overview of the syndecan-2 interactome, we combined the 30 novel MS partners (Table 1) together with the 41 literature partners identified in different tissues and species (Table 2) and grouped them according to the GO annotation biological process (Figure 4A). The MS partners (in green) distributed into several groups together with literature partners (Figure 4A), where the three largest were; cell communication, protein metabolism and cell growth and/or maintenance (Figure 4A). Within the three largest groups we also found the nine literature partners identified in our MS approach (Figure 4A underlined). We further performed a STRING database network analysis to predict connections among the 30 novel MS partners and the nine literature partners (Figure 4B). This revealed that the cardiac syndecan-2 interactome contained more connections than expected from a random set of proteins (PPI enrichment *p*-value: <0.000117, a list of all connections is given in Supplementary Table S2). Importantly, several of the novel MS partners connected with the literature partners and formed clusters (Figure 4B).

### Functional Annotation of the Syndecan-2 Interactome

The syndecan-2 interactome was also subjected to a functional annotation analysis through the DAVID Bioinformatics Resources^1^. The disease-class enrichment database revealed “cancer” and “cardiovascular” to be the top most prevalent enriched disease classes (Table 3). In line with this, the Kyoto Encyclopedia of Genes and Genome (KEGG) enrichment analysis predicted “proteoglycans in cancer,” “focal adhesion,” “leukocyte transendothelial migration,” “regulation of actin cytoskeleton,” and “pathways in cancer” as the top five pathways, but also “VEGF signaling” and different “cardiomyopathies” were predicted (Table 4). The DAVID tool was also used to search for enriched protein domains in the syndecan-2 interactome through the PFAM database (Table 5). The two most significant enriched protein domains were “integrin alpha” and” FG-GAP repeat,” which is part of the propeller structure of integrin alpha subunits. The third most enriched protein domain group was the “putative peptidoglycan binding domain,” which included several metalloproteinases. Interestingly, the fourth most enriched protein domain group was the CAVIN family, which contained the three novel MS partners caveolae-associated protein 1 (CAVIN1/PTRF), caveolae-associated protein 2 (CAVIN2/SDPR) and caveolae-associated protein 4 (CAVIN4/MURC).

**TABLE 3:** The genetic disease-class database analysis of the syndecan-2 interactome, 81.7% annotated.

Term	Gene name	Count	*p*-value
CANCER	*CXCL8, CXCR2, CDC42BPB, EPHB2, GIPC1, RASA1, SPHKAP, SRC, TIAM1, CDH1, CAV2, EZR, ITPR1, ITGA2, ITGAV, ITGB1, ITGB4, MMP14, MMP2, MMP7, MMP9, NF1, NOTCH3, PRKCA, PTPRJ, RAC1, RACK1, TGFBR1, TGFBR3, TRIM21*	30	1.7 × 10^–5^
CARDIOVASCULAR	*CAVIN4/MURC, CXCL8, CXCR2, FYN, RASA1, ARHGAP35, SPHKAP, TIAM1, CAV2, GPHN, ITPR1, ITGA2, ITGAV, ITGB1, JPH2, MMP14, MMP2, MMP7, MMP9, NF1, NOTCH3, PHACTR2, PRKCB, RAC1, REEP5, SDC2, SDC4, TGFBR1, TGFBR3*	29	2.0 × 10^–2^

**TABLE 4:** Enriched KEGG pathways in the syndecan-2 interactome, 70.4% annotated.

Term	Gene name	Count	*p*-value
Proteoglycans in cancer	*CAV2, CDC42, CTTN, EZR, ITPR1, ITGA2, ITGA5, ITGAV, ITGB1, MMP2, MMP9, PRKCA, PRKCB, PRKCG, RAC1, SDC2, SDC4, SRC, TIAM1*	19	5.1 × 10^–16^
Focal adhesion	*ARHGAP35, CAV2, CDC42, FYN, ITGA2, ITGA5, ITGAV, ITGB1, ITGB4, MYL12A, PRKCA, PRKCB, PRKCG, RAC1, SRC*	15	8.0 × 10^–11^
Leukocyte transendothelial migration	*ARHGAP35, CDC42, EZR, ITGAL, ITGB1, MMP2, MMP9, MYL12A, PRKCA, PRKCB, PRKCG, RAC1*	12	3.0 × 10^–11^
Regulation of actin cytoskeleton	*ARHGAP35, CDC42, EZR, ITGA2, ITGA5, ITGAL, ITGAV, ITGB1, ITGB4, MYL12A, RAC1, SRC, TIAM1*	13	1.6 × 10^–8^
Pathways in cancer	*CXCL8, CDH1, CDC42, ITGA2, ITGAV, ITGB1, MMP2, MMP9, PRKCA, PRKCB, PRKCG, RAC1, TGFBR1*	13	1.4 × 10^–5^
VEGF signaling pathway	*CDC42, PRKCA, PRKCB, PRKCG, RAC1, SRC*	6	6.6 × 10^–5^
Arrhythmogenic right ventricular cardiomyopathy (ARVC)	*ITGA2, ITGA5, ITGAV, ITGB1, ITGB4*	5	1.3 × 10^–3^
Hypertrophic cardiomyopathy (HCM)	*ITGA2, ITGA5, ITGAV, ITGB1, ITGB4*	5	2.2 × 10^–3^
Dilated cardiomyopathy	*ITGA2, ITGA5, ITGAV, ITGB1, ITGB4*	5	2.9 × 10^–3^
Vascular smooth muscle contraction	*ITPR1, PRKCA, PRKCB, PRKCG*	4	5.0 × 10^–2^
Viral myocarditis	*EIF4G3, FYN, ITGAL, RAC1*	4	7.6 × 10^–3^

**TABLE 5:** PFAM protein domains enriched in the syndecan-2 interactome, annotated 100%.

Term	Gene name	Count	*p*-value
Integrin alpha	*ITGA2, ITGA5, ITGAL, ITGAV*,	4	4.8 × 10^–5^
FG-GAP repeat	*ITGA2, ITGA5, ITGAL, ITGAV*	4	5.7 × 10^–5^
Putative peptidoglycan binding domain	*MMP2, MMP7, MMP9, MMP14*	4	6.7 × 10^–5^
PTRF/SDPR family	*CAVIN1/PTRF, CAVIN2/SDPR, CAVIN4/MURC*	3	9.4 × 10^–5^

### Relative Levels of the Syndecan-2 Interactome Partners in Cardiac Fibroblasts and Cardiomyocytes

**FIGURE 5:**
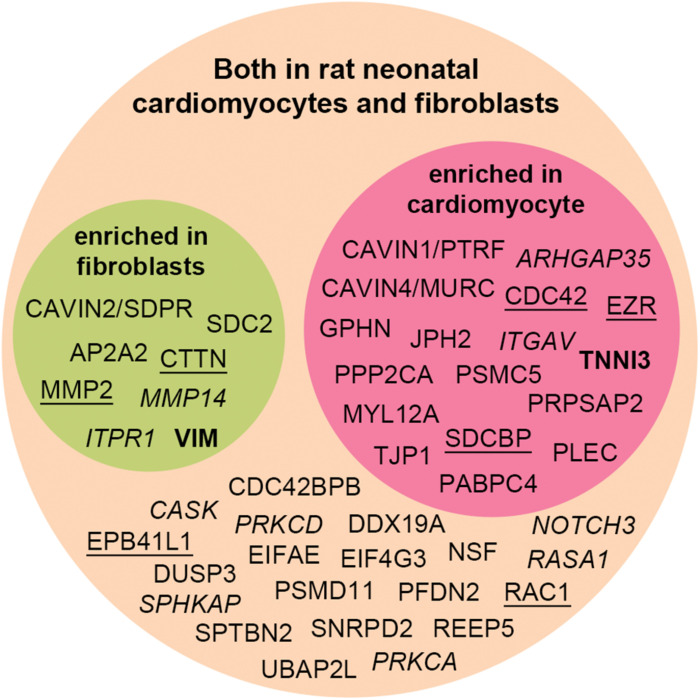
Distribution of the syndecan-2 interaction partners (novel and literature) in rat neonatal fibroblasts and cardiomyocytes identified by MS analyses. Literature partners also found in the AP-MS approach in this study are underlined, whereas literature partners we did not detect are in italic. Several proteins were significantly enriched in either fibroblast (green circle) or cardiomyocytes (red circle) and identified in both cell types (orange circle). VIM and TNNI3 are markers of fibroblasts and cardiomyocytes.

We decided to test whether the interactome proteins were mostly expressed in primary rat neonatal fibroblast or cardiomyocytes by MS analysis (Figure 5). Troponin-3 (TNNI3) was included as a marker for cardiomyocytes and vimentin (VIM) as a marker for fibroblasts (Figure 5 in bold). Purity of the cell fractions was based on the enrichment of TNN13 in the cardiomyocyte fraction and the lack thereof in the fibroblast fraction. Both endothelial cells and fibroblasts express vimentin [reviewed in Ivey and Tallquist (2016)] and some contamination of endothelial cells might have occurred. Except for NHLRC1, PHACTR2, RRAD, and TRIM21 (Table 1, marked^b)^), all novel MS partners and several of the syndecan-2 literature partners were identified in both cell types and most were enriched in one of the cell fractions (Figure 5). Syndecan-2 was enriched in the fibroblast fraction. The reason that not all interactome partners were detected might be because they are expressed at a more mature stage or expressed in other cell types.

## Discussion

In order to understand the mechanisms of cardiac disease, it is important to know the underlying players. Here we used three different AP-MS approaches to identify protein partners of the poorly described cardiac proteoglycan syndecan-2. In total, we identified 30 novel syndecan-2 partners and 9 out of 41 literature partners in rat LV lysates, which together constitute the first cardiac syndecan-2 interactome. Importantly, several of the interactome partners formed connections to each other, suggesting that these proteins are important for the role of syndecan-2 in the heart (Figure 4B).

Unlike a genome, a proteome is highly dynamic (Bonetta, 2010) and an interactome analysis like ours only provide a snapshot view of interaction partners in the given tissue. To include all potential syndecan-2 interaction partners, we used a membrane dissolving lysis buffer containing 1% triton to extract the LVs. We chose a relatively stringent set-up, where novel MS partners had to be detected in at least two of three AP-MS approaches. Although this strategy probably left out more transient binders, it increased the confidence in the novel syndecan-2 partners we identified in this study. Accordingly, all the novel partners tested in HEK293 cells showed binding to syndecan-2. Peptides of the syndecan-4 cytoplasmic domain have been shown to form dimers (Shin et al., 2001), but it is unsure if the syndecan-2 cytoplasmic tail without the transmembrane domain also form proper dimers (Choi et al., 2005). However, we identified both the C1 binder cortactin (CTTN) (Kinnunen et al., 1998) as well as the C2 binder syntenin-1 (SDCBP) (Grootjans et al., 1997) with the SDC2_cyt_ peptide, suggesting that the peptide did retain some functionality. However, to increase confidence in our results, we included a third AP approach using GST-SDC2, which successfully precipitated the C1 binder EZR (Granés et al., 2003). We detected more proteins in either pull down with biotin-ahx-SDC2_cyt_ or IP-GST-SDC2 than in both, which is probably due to the different nature of the baits.

Syndecans have generally been regarded as regulators of cell-matrix and cell-cell communication, and in particular, syndecan-2 has been coupled to dynamic processes and motile events (Oh and Couchman, 2004; Couchman et al., 2015). This was reflected in the syndecan-2 interactome where the largest group was “cell communication” (Figure 4A), the top disease enrichment was “cancer” and “cardiovascular” (Table 3), and the top KEGG pathway was “proteoglycans in cancer” (Table 4). In line with this, the largest cluster of connected proteins was involved in cytoskeletal remodeling and migration (Figure 4B, main cluster). One of these novel partners was the AP-2 complex subunit α-2 (AP2A2), which connected with the two literature partners CTTN and EZR (Figure 4B, cluster 1). AP2A2 is an adaptor molecule involved in endocytosis of cargo and has been shown to mediate endocytosis of the syndecan-2 co-receptor CXCR2 (Renga et al., 2012; Raman et al., 2014). It also coordinates intracellular trafficking together with ARF6, a GTP binding protein found to regulate intracellular traffic of the syndecans, along with the literature partner SDCBP (Zimmermann et al., 2005; Lau and Chou, 2008). EZR links the plasma membrane to the actin cytoskeleton and has been found to regulate podosomal rosette formation together with CTTN in pancreatic cancer cells (Kocher et al., 2009). The rosette structures have been associated with invasive properties and reported to require adhesion to fibronectin (FN) and subsequent digestion of the ECM (Kocher et al., 2009). CTTN has been shown to regulate the secretion of FN, which is necessary for cell motility (Sung et al., 2011; Schnoor et al., 2018) and is also reported to regulate secretion of ECM digesting matrix metalloproteinases (MMPs) (Clark et al., 2007), which were also present in the syndecan-2 interactome (Figure 4B, main cluster). Interestingly, knockdown of syndecan-2 in fibroblasts has been shown to block FN matrix assembly (Galante and Schwarzbauer, 2007) and cells expressing syndecan-2 without the cytoplasmic tail have been found unable to assemble matrix at the cell surface (Klass et al., 2000) and to form proper FN fibrils in *Xenopus* (Kramer and Yost, 2002). Because of the involvement of CTTN in FN secretion, it is tempting to speculate that CTTN might act prior to the FN assembly role of syndecan-2. Overall, this points to a role for syndecan-2 in regulating cortical actin dynamics, possibly mediated through trafficking of co-receptors, cargo or syndecan-2 itself. AP2A2, CTTN and syndecan-2 were all found enriched in fibroblast fractions (Figure 5). Since ECM secretion is a characteristic feature of myofibroblasts [reviewed in Frangogiannis (2019)] and syndecan-2 has been shown to cooperate with integrin β1 (ITGB1) (Figure 4B, main cluster) in stress fiber formation (Kusano et al., 2000), another myofibroblast feature, we speculate whether this cluster of proteins might play a role in cardiac fibroblast activation.

The two novel MS partners serine/threonine-protein kinase MRCKβ (CDC42BPB) and tight junction protein ZO-1 (TJP1) connected to the syndecan-2 literature partner cell division control protein 42 homolog (CDC42) (Figure 4B, cluster 2). CDC42 is a plasma membrane associated small GTPase involved in syndecan-2 mediated filopodia extensions (Granes et al., 1999), and CDC42BPB is a CDC42 effector kinase (Huo et al., 2011). After binding of CDC42, CDC42BPB has been shown to form a complex with TJP1, which targets the CDC42BPB-TJP1 complex to the leading edge of migrating cells (Huo et al., 2011). TJP1 is a scaffolding protein that has been found to bind to cell surface transmembrane receptors through its N-terminal PDZ domain and further couples them to the actin cytoskeleton through its C-terminal proline rich region (Fanning et al., 1998; González-Mariscal et al., 2000). Although initially identified in tight junctions, TJP1 has also been found to regulate dynamic processes like angiogenesis and migration (Mattagajasingh et al., 2000; Tornavaca et al., 2015). Since syndecan-2 spans the plasma membrane, we speculate whether syndecan-2 is involved in the membrane localization of the CDC42BPB-TJP1 complex, and perhaps these proteins work together in cytoskeletal rearrangements and/or angiogenesis. TJP1 also connects to the MS partner plectin (PLEC) (Figure 4B, main cluster), which has been shown to be important in vascular integrity and to dysregulate tight junctions when absent (Osmanagic-Myers et al., 2015). Less is known about the novel MS partner myosin regulatory light chain 12A (MYL12A) (Figure 4B, main cluster), but it has been suggested to be involved in fibroblast contractility (Park et al., 2011).

Protein metabolism is important during cardiac remodeling (Chorghade et al., 2017). Interestingly, several MS partners were involved in protein metabolism, including the eukaryotic initiation factor 4E (EIF4E) and prefoldin subunit 2 (PFDN2) (Figure 4B, cluster 3). EIF4E is involved in regulation of translation initiation [reviewed in Shahbazian et al. (2006) and Siddiqui and Sonenberg (2015)] and PFDN2 is a co-chaperone involved in folding of cytosolic protein [reviewed in Sahlan et al. (2018)]. Like the well-known syndecan partner SDCBP (Grootjans et al., 1997), both EIF4E and PFDN2 precipitated in all three AP-MS approaches (Figure 2), which suggested that these bind syndecan-2 quite robustly. The third cluster was connected to the main cluster through the novel partner serine/threonine-protein phosphatase 2A catalytic subunit α (PPP2CA), which is known to regulate multiple cardiac signaling pathways [reviewed in Lubbers and Mohler (2016)] and to have a vast amount of targets, including EIF4E (Li et al., 2010). We cannot exclude that PPP2CA might also dephosphorylate syndecan-2. Although dephosphorylation of syndecans is less well understood, we have previously found that dephosphorylation of syndecan-4 might work as a molecular switch in the progression toward heart failure (Finsen et al., 2011). Future studies are needed to investigate the role of syndecan-2 in protein metabolism.

The last cluster consisted of three novel syndecan-2 partners, all from one of the most abundant families in the interactome, the CAVIN family (Table 5 and Figure 4B, cluster 4). Members of the CAVIN family have been reported to form homo- and heterologous complexes at caveolae sites, where CAVIN1 and CAVIN2 are involved in caveolae formation and curvature (Hill et al., 2008; Hansen et al., 2009; Nassar and Parat, 2015). In addition, individual signaling roles have been reported for all members. In response to insulin like growth factor 1 (IGF-1) and in consort with caveolin-1 (CAV1), CAVIN1 has been reported to regulate endocytosis and thereby signaling of the IGF-1 receptor (Aboulaich et al., 2006; Salani et al., 2010). IGF-1 has also been proposed to regulate syndecan-2-mediated actin polymerization and migration of a fibroblast cell line (Mytilinaiou et al., 2017). Moreover, in lungs, syndecan-2 has been reported to be involved in the sequestering of pro-fibrotic TGF-β1 receptors into intracellular vesicles together with CAV1 (Shi et al., 2013; Tsoyi et al., 2018). Taken together this could suggest that syndecan-2 might mediate the binding of growth factors, like IGF-1, to co-receptors at caveolae sites, leading to internalization in cooperation with the CAVINs and CAV1. Since, e.g., insulin has been suggested to mediate translocation of CAVIN1 (Liu and Pilch, 2016), it is also possible that syndecan-2 in response to growth factor stimulation, regulates the release of CAVINs from the membrane. Less is known about CAVIN2, but like syndecan-2, CAVIN2 has been found to be upregulated after aortic banding (Ogata et al., 2008), able to regulate angiogenesis in zebrafish (Chen E. et al., 2004; Ogata et al., 2008; Boopathy et al., 2017) and enriched in cardiac fibroblasts (Figure 5). Future studies will reveal the role of their interaction in cardiac fibroblasts and if CAVIN2 has a role in the syndecan-2 mediated angiogenesis. The CAVIN1 knock-out mouse shows cardiac dysfunction including fibrosis (Taniguchi et al., 2016), whereas CAVIN2 has not yet been coupled to fibrosis. CAVIN4 is restricted to muscle cells and involved in cardiac dysfunction (Ogata et al., 2008; Ogata et al., 2014). Overall, this could point to a role for syndecan-2 and the CAVINs in heart disease.

**FIGURE 6:**
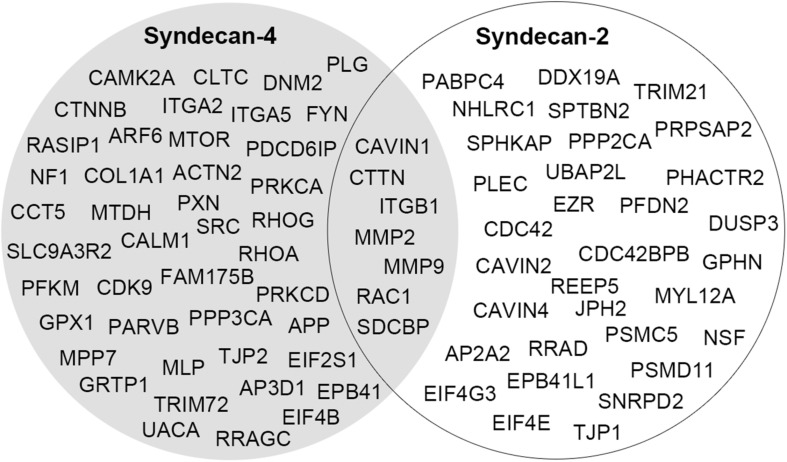
A comparison of the cardiac interactomes of syndecan-2 (white circle) and the syndecan-4 (Mathiesen et al., 2019) (gray circle).

In two recent studies, syndecan-2 interaction partners were extracted from various interactome databases and previous high-throughput AP-MS experiments (Huttlin et al., 2015; Gondelaud and Ricard-Blum, 2019; Zandonadi et al., 2019). These did not overlap with the novel MS partners identified in our study and is probably a consequence of the different AP methods, tissues and cell models used. Recently we also identified the cardiac syndecan-4 interactome using similar strategy as described here (Mathiesen et al., 2019). Despite the close relationship between syndecan-2 and syndecan-4, their cardiac interactomes were surprisingly different, suggesting different functions. Except for the literature partners CTTN, ITGB1, MMP2, MMP9, RAC1, and SDCBP, we only found the novel MS partner CAVIN1 to be a common partner (Figure 6). The two syndecans also connected to some proteins within the same family, including the adaptor protein complexes (AP2A2 and AP3D1), tight junction proteins (TJP1 and TJP2) and the protein 4.1 proteins (EPB41L1 and EPB41) (Figure 6). Notably, proteins that bind to the common C1 or C2 region are often listed to bind all four syndecans (Gondelaud and Ricard-Blum, 2019; Mathiesen et al., 2019) (Table 2). However, the relatively low overlap of the C1 and C2 binding partners in the cardiac syndecan-2 and syndecan-4 interactomes suggests that this might not always be the case. On the other hand, CTTN has previously only been described as a syndecan-3 partner, but its presence in both the syndecan-2 and syndecan-4 interactomes suggests that it can indeed bind multiple syndecans.

All four syndecans are expressed in the heart (Strand et al., 2013), however, previous studies have focused on syndecan-1 and -4. Syndecan-1 is mainly known as a pro-fibrotic player and regulator of immune cell infiltration after a myocardial infarct (Vanhoutte et al., 2007; Frangogiannis Nikolaos, 2010; Schellings Mark et al., 2010). Syndecan-4 has been shown to act as a pro-remodeling molecule in both cardiomyocytes and fibroblasts in addition to recruiting immune cells (Finsen et al., 2011; Herum et al., 2013, 2015; Strand et al., 2013). Based on our data, the notion that the expression of syndecan-2 increases after aortic banding (Strand et al., 2013) and that syndecan-2 has been correlated with fibrosis in other tissues (Chen L. et al., 2004; Renga et al., 2012; Ruiz et al., 2012) it is tempting to speculate that syndecan-2 might be involved in cardiac fibrosis, perhaps together with CTTN and the CAVINs. Another important observation is that the interactome partners are not necessarily involved in cell adhesion, which is a primary feature associated with syndecans, thus the main role of syndecan-2 in the heart might be found outside the major adhesion sites.

Future studies are needed to verify the syndecan-2 interactions identified in this study and to determine their biological significance. Altogether, we hope that the interactome will spur future hypotheses and direct future studies on syndecan-2 in the heart.

## Materials and Methods

### Antibodies

Immunoprecipitations and immunoblotting were carried out using anti-SDC2 (LS-C150258, Nordic Biosite, Sweden), normal sheep IgG (6C0333, Merck KGaA, Germany), anti-GST (sc-80998, Santa Cruz Biotechnology, Inc., United States), anti-FLAG (F1804, Sigma-Aldrich, United States), anti-HA (#3724, clone C29F4, Cell Signaling, Netherlands) and anti-biotin-HRP (A0185, Sigma-Aldrich, United States). HRP conjugated anti-sheep (6150-05, SouthernBiotech, United States), anti-mouse (NA931V, GE Healthcare, United States) and anti-rabbit (NA934V, GE Healthcare) were used as secondary antibodies.

### Peptides and Recombinant Proteins

Customized peptides were synthesized to >80% purity by Genscript Corp. (United States): Biotin-ahx-SDC2_cyt_: RMRKKDEGSYDLGERKPSSAAYQKAPTKEFYA and biotin-ahx-scrambled (scram): RLEDKRPAQAKGKATMESFYKYDPRSAYGESK. Recombinant GST and N-terminal GST-tagged SDC2 (mouse) proteins were also made by Genscript Corp.

### Transfection of HEK293 Cells

Human Embryonic Kidney 293 (HEK) cells (ATCC CRL-1573™, United States) were kept in Dulbecco’s modified Eagles’s medium (DMEM) (41965-039, Gibco, Life Technologies, Inc., United States) supplemented with 10% fetal bovine serum (FBS) and 1% penicillin/streptomyocin (PS) (P0781, Sigma-Aldrich) humidified at 37°C in 5% CO_2_. HEK293 cells were transfected with the CaCl_2_ method as previously described (Jordan et al., 1996; Mathiesen et al., 2019). Briefly, cells were cultured without PS 24 h before transfection. Plasmid DNA (8 μg) in 500 μL CaCl_2_ solution (248 mM) was mixed with 500 μL 2x HEPES buffer (50 mM HEPES, 280 mM NaCl, 1.5 mM Na_2_HPO4, pH 7.0), incubated at room temperature (RT) for 20–30 min (min) before drippled onto the cells. After 24 h cells were harvested in IP buffer [150 mM NaCl, 20 mM HEPES (pH 7.5), 1 mM EDTA, 1%Triton X-100] with cOmplete protease inhibitor cocktail (#05050489001, Roche, Switzerland). All genes were cloned with either a FLAG or HA tag in the pcDNA3.1 vector unless otherwise stated (Genscript Corp., United States). The DNA constructs were: DDX19A-FLAG human (NM_018332), NSF-FLAG human (NM_006178), PFDN2-FLAG human (NM_012394), PSMC5-FLAG human (NM_001199163), PSMD11-FLAG mouse (NM_178616), MYL12A/RLC-A-FLAG rat (NM_001135017), CAVIN2/SDPR-FLAG human (NM_004657), CTTN-FLAG human (NM_138565), EIF4E-FLAG human (NM_001968), JPH2-FLAG human (NM_020433.4), RRAD-FLAG human (NM_001128850), 3x FLAG-HSPB6 rat (NM_138887.1), FLAG-His6-PPP1CA rat (P62138) and HA-SDC2 human (NM_002998) (pCEP4 vector). AP2A2-FLAG human (NM_001242837.1) was cloned by Cyagen US Inc. (United States). The 11 interactors were selected based on a combination of their distribution in the pull down, IP-GST and IP-SDC2 AP-MS groups, distribution in the cardiac syndecan-2 interactome (Figure 4B) as well as availability from the Genscript clone collection.

### Immunoprecipitation (IP) in Cell Lysates

Lysates were mixed with 2 μg anti-FLAG, anti-SDC2 or anti-GST (with recombinant GST-SDC2) and protein A/G-agarose beads (sc-2003, Santa Cruz Biotechnology) and rotating overnight (ON) at 4°C. After three times wash in IP buffer (150 mM NaCl, 20 mM HEPES, pH 7.5, 1 mM EDTA, 1%Triton X-100) with cOmplete protease inhibitor cocktail (#5050489001, Roche), samples were eluted by boiling in 2 × SDS loading buffer and analyzed by immunoblotting.

### Immunoblotting

Samples were analyzed on 4–15% or 15% Criterion™ Tris-HCl precast gel (#3450028 and #3450021, Bio-Rad, United States) and blotted onto a PVDF membrane (#1704157, Bio-Rad). After blocking in either 5% non-fat dried milk or 1% casein in TBS-T [Tris-buffered saline with 1% Tween-20 (#1610781, Bio-Rad)] for 1 h at RT, membranes were incubated with primary antibodies for 1 h at RT or ON at 4°C. Following three times 5 min wash in TBS-T, membranes were incubated with HRP-conjugated secondary antibody for 1 h at RT, washed three times 5 min in TBS-T and signal developed using ECL Prime (RPN 2232, GE Healthcare). Reprobing was performed after stripping with Restore™ Western Blot Stripping Buffer (#21059, Thermo Scientific, United States).

### Peptide Overlay

Syndecan-2 (rat, mouse, and human) and syndecan-4 (rat) were spot-synthesized as 20-mer peptides with 3 amino acids offset on a cellulose membrane by a Multiprep automated peptide synthesizer (INTAVIS Bioanalytical Instruments AG, Germany) (Frank and Overwin, 1996). The peptide array membranes were blocked for minimum 1 h in 1% casein in TBS-T at RT before incubation with anti-syndecan-2 ON at 4°C. Binding was detected by immunoblotting with anti-sheep-HRP and signal detected by ECL Prime.

### Rat Neonatal FB and CM

The study conforms to the “Guide for the Care and Use of Laboratory Animals” (NIH publication No. 85-23, revised 2011, United States) and was preapproved by the Norwegian National Animal Research Committee (Permit of approval number IV1-17U). Lysates from primary cardiomyocytes and fibroblasts were prepared as previously described (Mathiesen et al., 2019) and thereafter analyzed by MS.

### LV Lysate and Affinity Purification for MS

Frozen LV’s from Wistar 230-250 g male rats (Janvier Labs, France) were pulverized in liquid nitrogen in a mortar, transferred to lysis buffer [150 mM NaCl, 20 mM Hepes, pH 7.5, 1 mM EDTA, 0.5% Triton supplemented with complete protease inhibitor cocktail (Roche)] on ice and homogenized with a Polytron 1200 homogenizer in three series of 1 min. The suspensions were centrifuged at 100,000 × *g* for 60 min at 4°C and supernatants were stored at −80°C.

In pull down experiments with peptides, pooled LV lysates were mixed with 0.01 mM of the biotinylated SDC2_cyt_ peptide or 0.02 mM of the biotinylated scrambled control peptide (to secure excess of negative control) and rotated ON at 4°C. LV lysates without any peptides was included as a second negative control (beads only). Streptavidin coated dynabeads (Dynabeads™ M-270 Streptavidin, #65305, Life Technologies, United States) were washed three times in PBS before they were added to the LV lysate with or without peptides and rotated for 40 min at RT. The beads were washed five times in PBS and captured proteins were eluted in 250 μL 25 mM biotin for 3 h at 60°C. Proteins were precipitated in 1 ml of 4 × ice-cold acetone added glycoblue at −20°C ON. The tubes were centrifuged and the pellets were air-dried before MS analysis.

For large scale IP, 10 μg/mg of anti-syndecan-2, anti-sheep IgG or anti-GST were coupled to magnetic dynabeads (Dynabeads™ Antibody Coupling Kit, #14311D, Thermo Fisher, United States) according to manufacturer’s protocol. The antibody coupled beads were incubated with the LV lysates with or without supplementation of GST or GST-SDC2 recombinant protein and rotated ON at 4°C. After three times wash in cold PBS and two times wash in cold water to remove salts (Mlynarcik et al., 2012), captured protein complexes were eluted in 0.1% TFA in freshly made 50% acetonitrile for 30 min while rotating at RT. The elution step was repeated once with fresh TFA before precipitation in 1 ml 4 × ice-cold acetone added glycoblue at −20°C. Samples were spun at max speed for 15 min and the pellets were air-dried before MS analysis.

### Protein Identification and Label-Free Quantification by LC-MS/MS

The 2-D Clean Up-Kit (80-6484-51, GE healthcare) was used to precipitate proteins from the fibroblast and cardiomyocyte fractions. The precipitate was then dissolved in 40 μL 0.2% ProteaseMAX™ Surfactant, Trypsin Enhancer (Promega) in 50 mM NH_4_HCO_3_ before protein reduction, alkylation and in-solution trypsin digestion (Promega) ON at 37°C. Following digestion, centrifugation at 14,000 × *g* for 10 min, trypsin inactivation by adding 100 μl 1% TFA and another round of centrifugation at 14,000 × *g* for 10 min followed.

The air dired IP samples were resuspended in 20 μl 6 M Urea in 10 mM HEPES, pH 8 before reduction, alkylation and 4 h in-solution lysyl endopeptidase digestion (Wako) in room temperature. Samples were diluted four times before over night trypsin (Promega) digestion at room temperature. Following digestion, centrifugation at 14,000 × *g* for 10 min, trypsin inactivation by adding 100 μl 1% TFA and another round of centrifugation at 14,000 × *g* for 10 min followed.

Desalting and upconcentration of peptides was performed before MS by the STAGE-TIP method using a C18 resin disk (3M Empore). The peptides were eluted with 80 μl 80% ACN/0.1% FA, dried, and solubilized in 7 μL 0.1% FA for MS analysis. Each peptide mixture was analyzed by a nEASY-LC coupled to QExactive Plus (Thermo Electron, Bremen, Germany) as previously described (Mathiesen et al., 2019), except that a 50 cm column was used. Gradients of 60 and 120 min were used for the IPs and fractions, respectively. The resulting MS raw files were submitted to the MaxQuant software for protein identification and label-free quantification and Perseus software was used for the statistical analysis as described in detail previously (Mathiesen et al., 2019).

To allow quantitative comparisons and determine the significance between MS samples, LFQ intensities were loaded into Perseus (Version 1.4.0.20), log2 transformed and a *t*-test was performed (*p* < 0.05 was accepted as statistically significant). To be considered a MS partner, proteins had to be identified in IP-SDC2 and at least one of the other APs. Non-specific binders to the SDC2_cyt_ peptide were sorted away through the two negative controls; scramble or beads only. Non-specific binders to GST-SDC2 were sorted away through the two negative controls; GST or beads only. Then the pools of proteins identified in the pull down and IP-GST were compared with the pool of proteins obtained from IP-SDC2 corrected against IP-IgG.

### Generating the Cardiac Interactome Map

The 30 MS partners and 41 literature partners were grouped according to the GO annotation biological process extracted from hprd.org (Keshava Prasad et al., 2009) (Figure 4A). The MS partners and literature partners found in our AP-MS screens (this study) (Figure 2) made up the cardiac interactome and STRING database version 10.5 (Szklarczyk et al., 2017) was used to predict connections (Figure 4B). Data was extracted with the following setting: species: *Homo sapiens* (created more connections than *Rattus norvegicus*), confidence: medium, active interaction sources: Experiments, databases, and co-expression. The network stats were: number of nodes: 39, number of edges: 48, average node degree: 2.46, avg. local clustering coefficient: 0.395, expected number of edges: 27. PPI enrichment *p*-value: <0.000117. CAVIN4 has been shown to interact with CAVIN1 and -2 in the literature (Ogata et al., 2008, 2014) and was therefore added to the interactome. The literature partners CDC42, ITGB1, EZR, MMP2, MMP9, RAC1, and EPB41L1 were detected in only one AP-MS approach. However, since they were identified both in this and in another study (Table 2), their potential for being important for syndecan-2 in the heart was heightened, and they were therefore included in the STRING analysis.

### Functional Annotation

The DAVID bioinformatics tool (Huang da et al., 2009a, b), version 6.8, was used for functional annotation. Through the DAVID tool enrichment in genetic disease-class database annotation, KEGG pathways, and PFAM (Finn et al., 2016) domains were analyzed. The SDC2 interactome gene list was imported as official gene names and *Homo sapiens* was used as both species and background and the following settings were used count (2) and EASE score (0.1) (Modified Fisher Exact *p*-value).

## Data Availability Statement

The datasets generated in this study can be found in online repositories. The names of the repository/repositories and accession number(s) can be found below: http://www.proteomexchange.org/, PXD018942.

## Ethics Statement

The animal study was reviewed and approved by Norwegian National Animal Research Committee.

## Author Contributions

SM and CC designed and conceived the experiments. SM wrote the manuscript under the guidance of CC and GC. SM performed the largescale AP. MS and TN performed the mass spectrometry analysis. MM performed the transfections. SM and ML performed the small scale IPs and epitope mapping. All authors read and approved the manuscript.

## Conflict of Interest

The authors declare that the research was conducted in the absence of any commercial or financial relationships that could be construed as a potential conflict of interest.

## Footnotes

1 https://david.ncifcrf.gov/

## Abbreviations

AP: affinity purification
AP-MS: affinity purification coupled to mass spectrometry
ECM: extra cellular matrix
GST: glutathione *S*-transferase
HRP: horseradish peroxidase
IP: immunoprecipitation
literature partner: syndecan-2 interaction partner previously described in the literature
LV: left ventricle
MS partner: syndecan-2 interaction partner identified in AP-MS in this study
MS: mass spectrometry
Scram: scrambled peptide
SDC2: syndecan-2
SDC2_cyt_: cytoplasmic domain of syndecan-2.
