# Supplementary material for: The Cardiac Syndecan-2 Interactome
Source: Front Cell Dev Biol. 2020 Aug 28;8:792. doi: 10.3389/fcell.2020.00792 (PMC7483480; doi:10.3389/fcell.2020.00792)
Supplement: Supplementary file 3 [file Data_Sheet_1.PDF]

Uncropped western blots for Figure 1D

IP-SDC2 IP-IgG

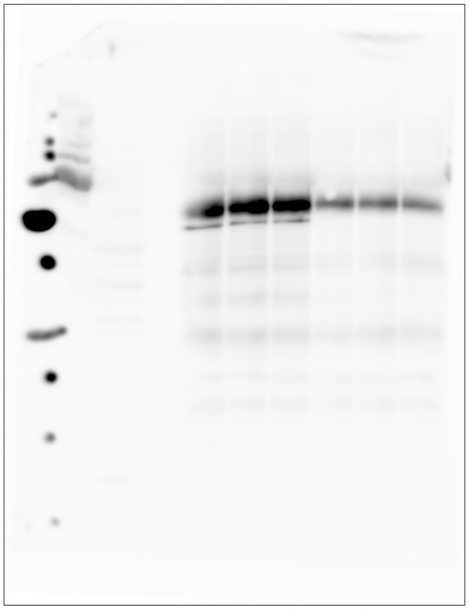

anti-SDC2

Uncropped western blots for Figure 1E

input IP-GST

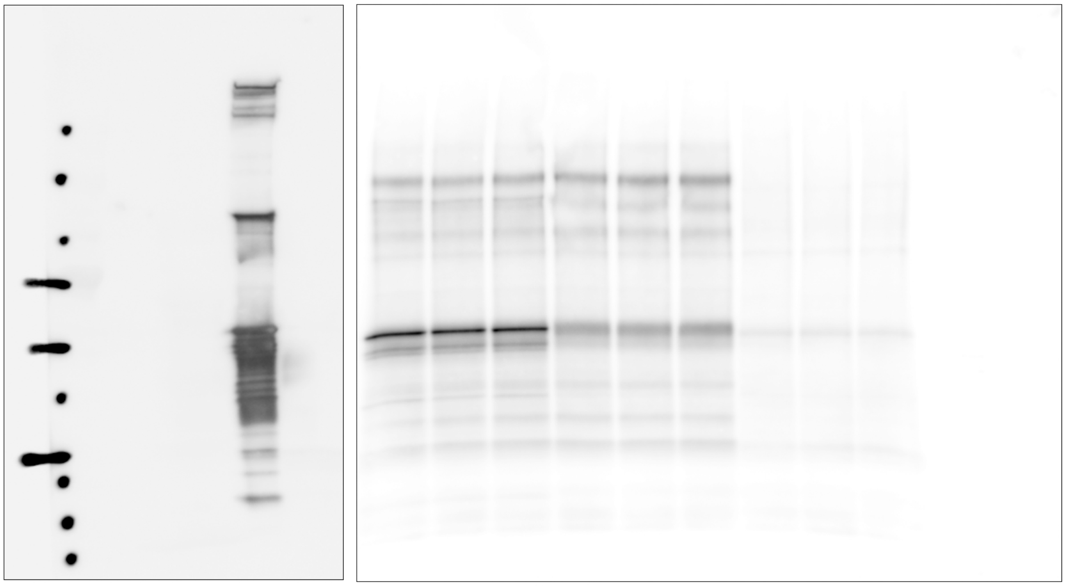

anti-SDC2

Uncropped western blots for Figure 3A

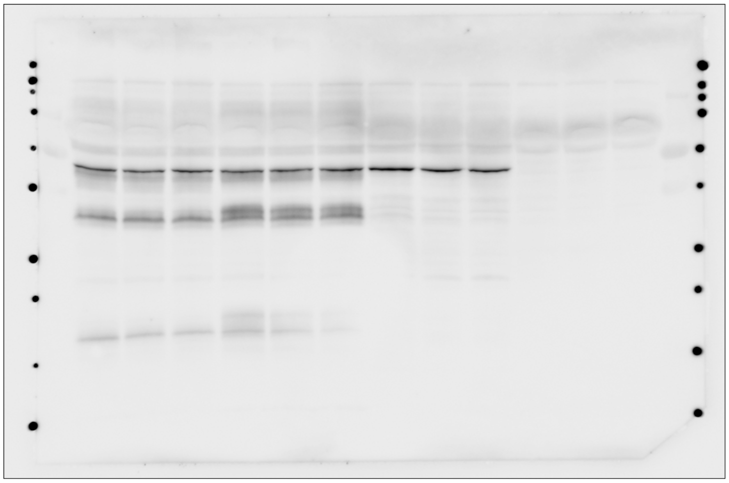

anti-HA

Uncropped western blots for Figure 3B

IP-FLAG

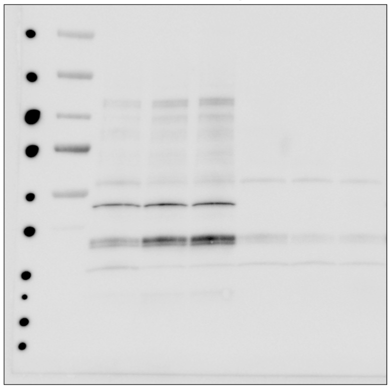

anti-HA

IP-FLAG

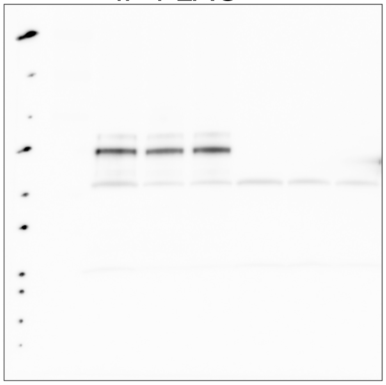

anti-FLAG

Lysates

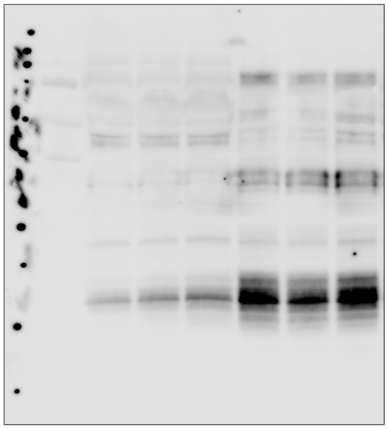

anti-HA

Lysates

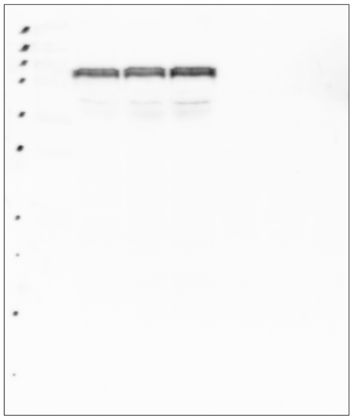

anti-FLAG

Uncropped western blots for Figure 3C

IP-FLAG

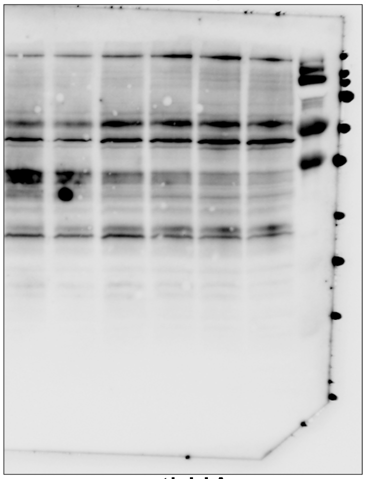

anti-HA

IP-FLAG

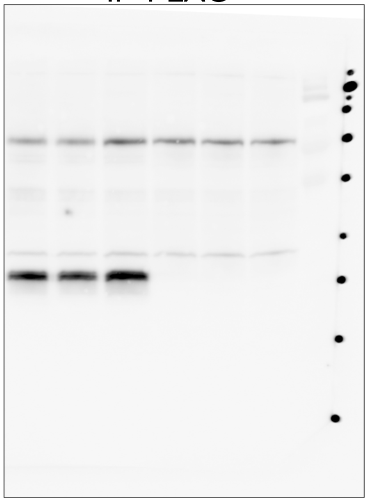

anti-FLAG

Lysates

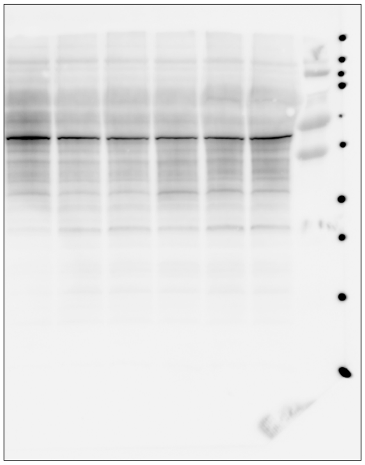

anti-HA

Lysates

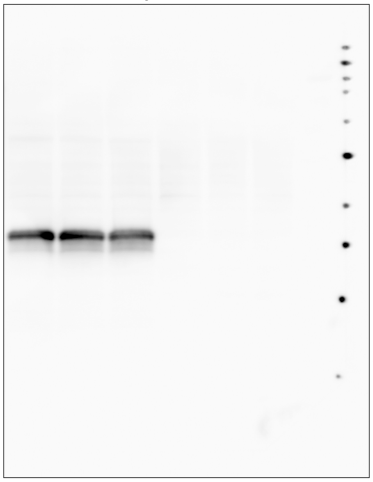

anti-FLAG

Uncropped western blots for Figure 3D

IP-FLAG

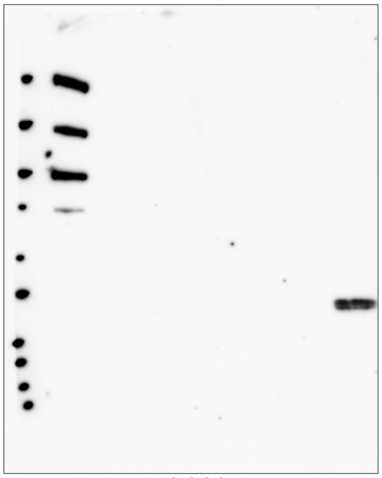

anti-HA

IP-FLAG

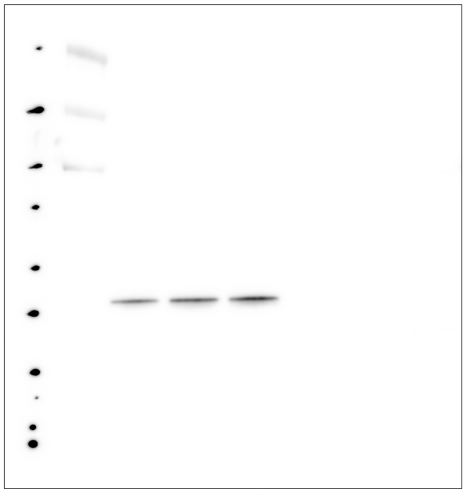

anti-FLAG

Lysates

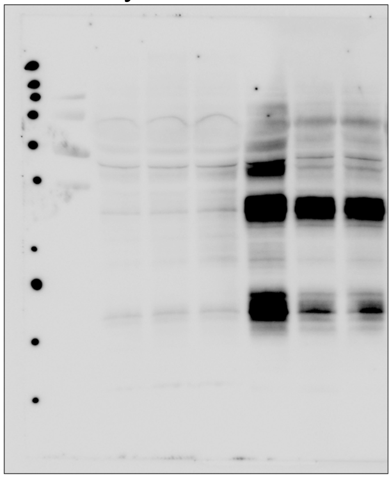

anti-HA

Lysates

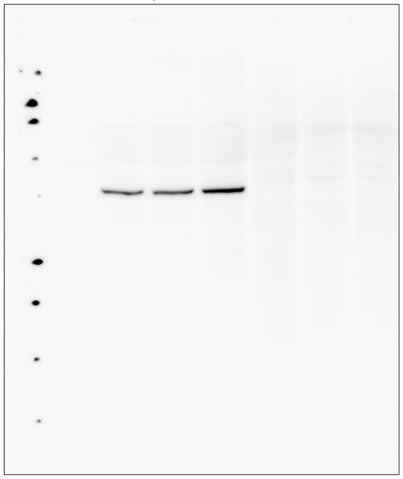

anti-FLAG

Uncropped western blots for Figure 3E

IP-FLAG

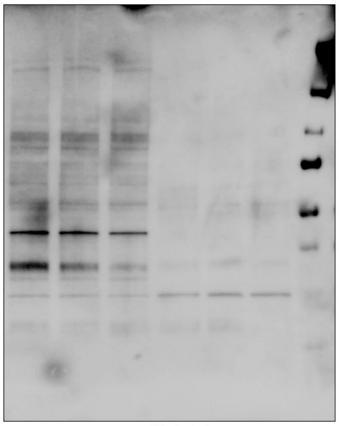

anti-HA

IP-FLAG

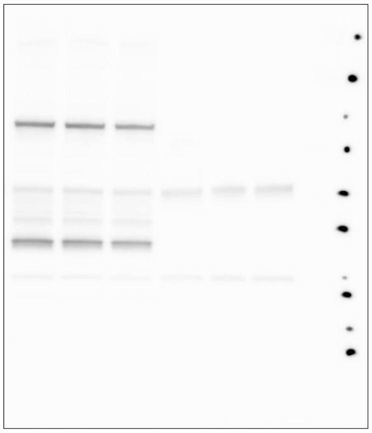

anti-FLAG

Lysates

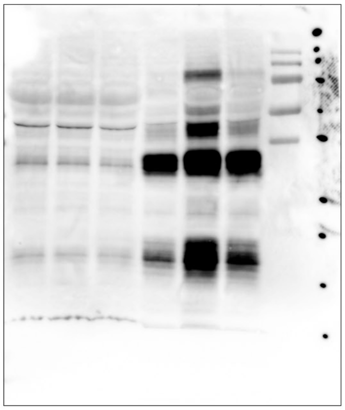

anti-HA

Lysates

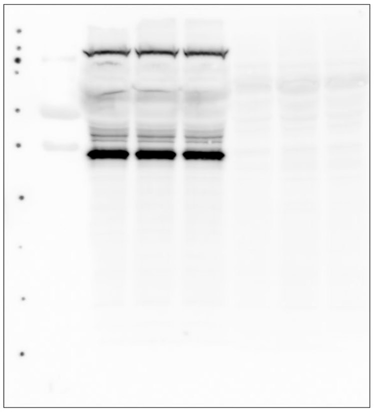

anti-FLAG

Uncropped western blots for Figure 3F

IP-FLAG

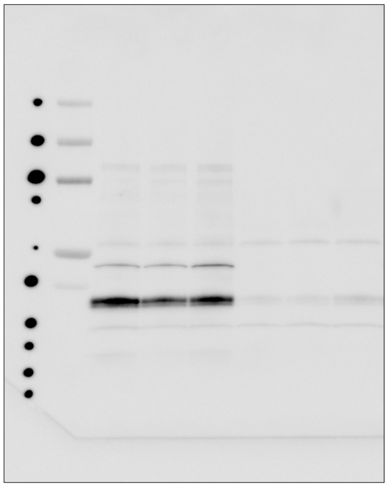

anti-HA

IP-FLAG

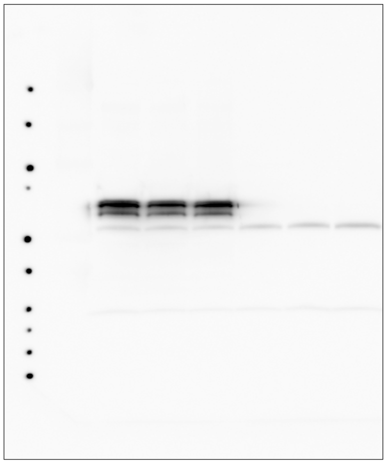

anti-FLAG

Lysates

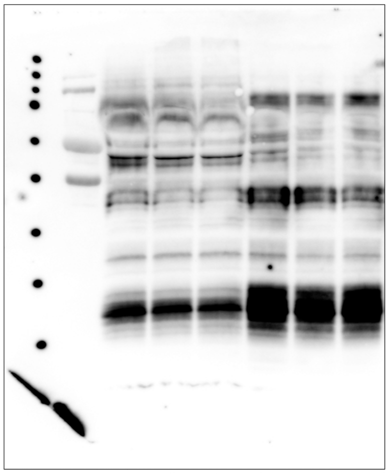

anti-HA

Lysates

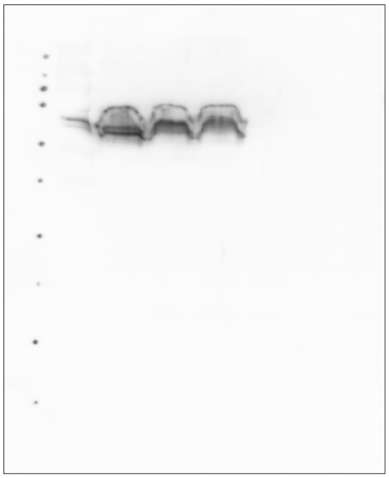

anti-FLAG

Uncropped western blots for Figure 3G

IP-FLAG

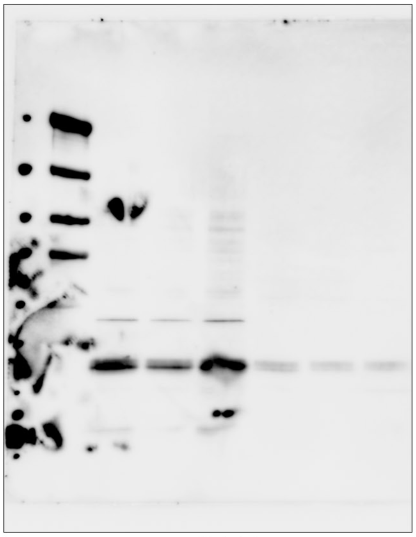

anti-HA

IP-FLAG

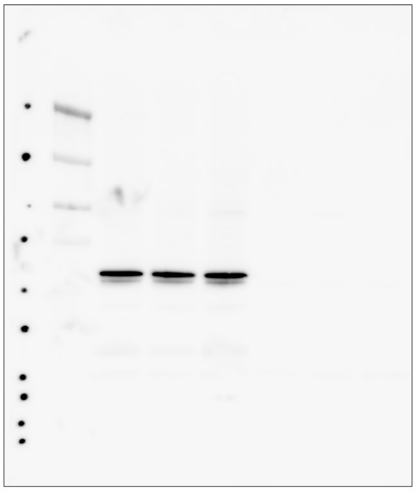

anti-FLAG

Lysates

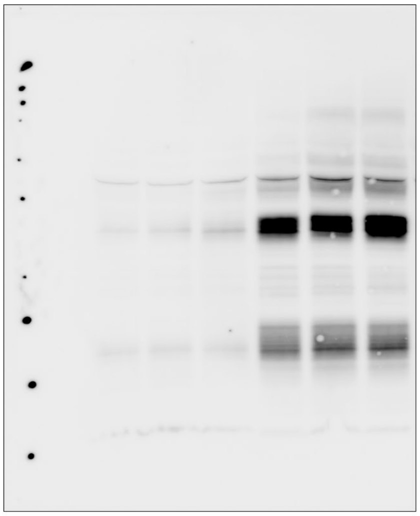

anti-HA

Lysates

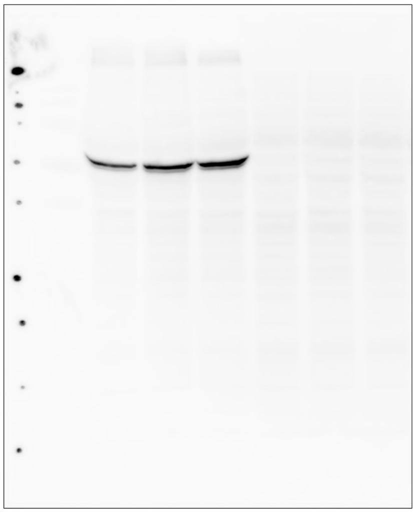

anti-FLAG

Uncropped western blots for Figure 3H

IP-FLAG

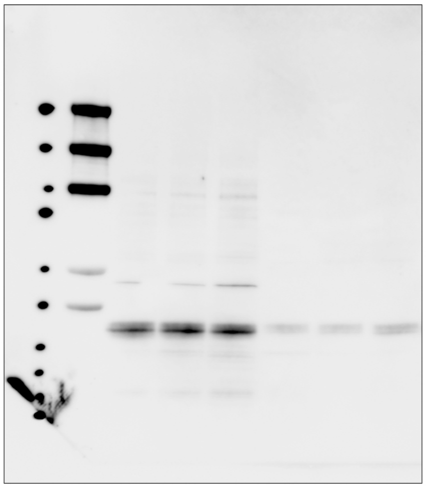

anti-HA

IP-FLAG

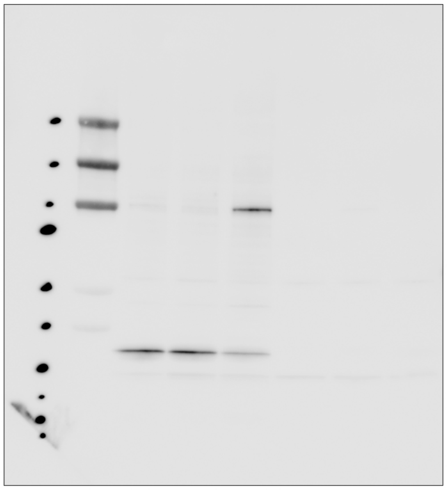

anti-FLAG

Lysates

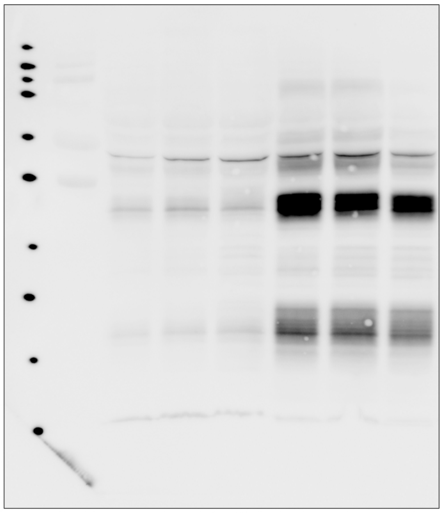

anti-HA

Lysates

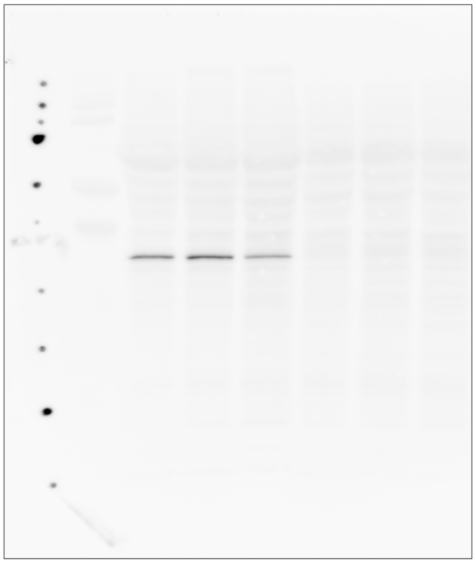

anti-FLAG

Uncropped western blots for Figure 3I

IP-FLAG

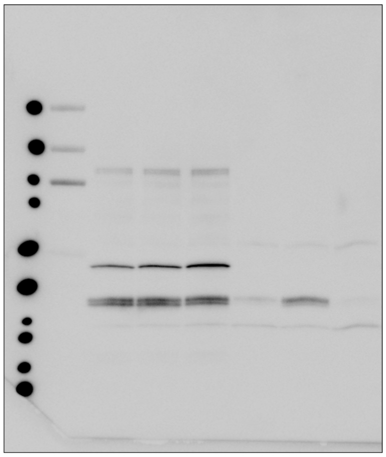

anti-HA

IP-FLAG

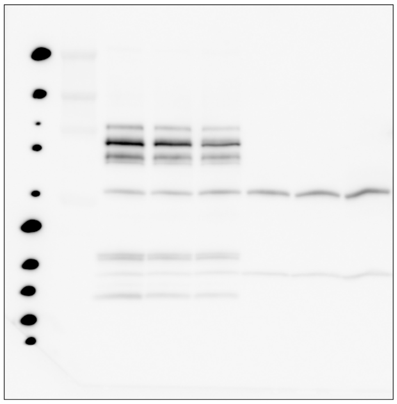

anti-FLAG

Lysates

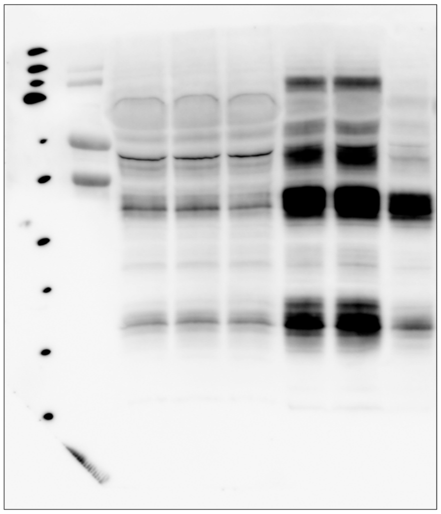

anti-HA

Lysates

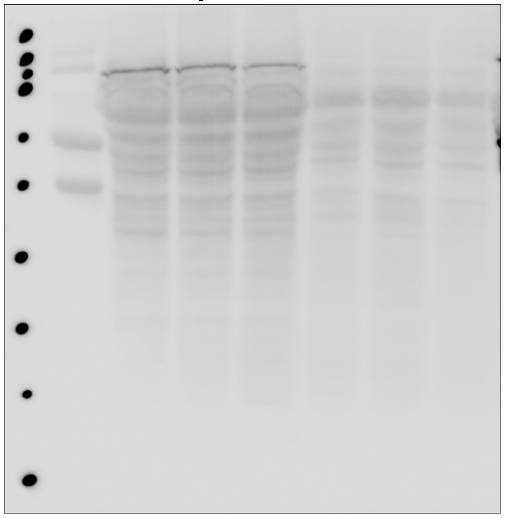

anti-FLAG

Uncropped western blots for Figure 3J

IP-FLAG

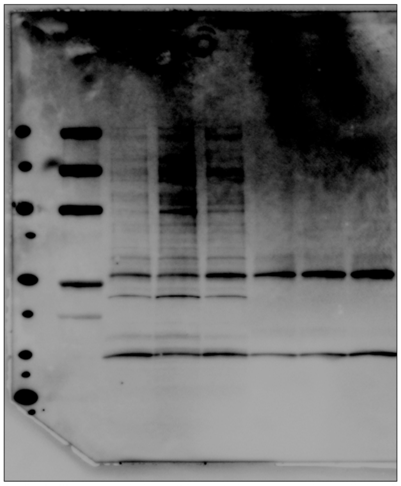

anti-HA

IP-FLAG

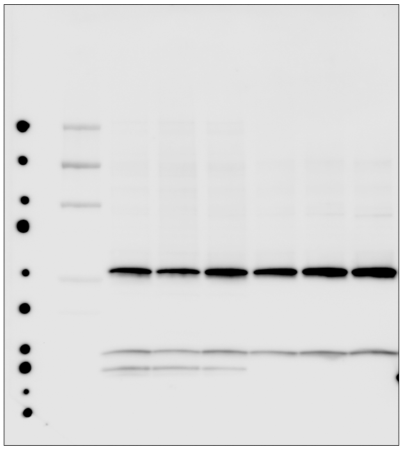

anti-FLAG

Lysates

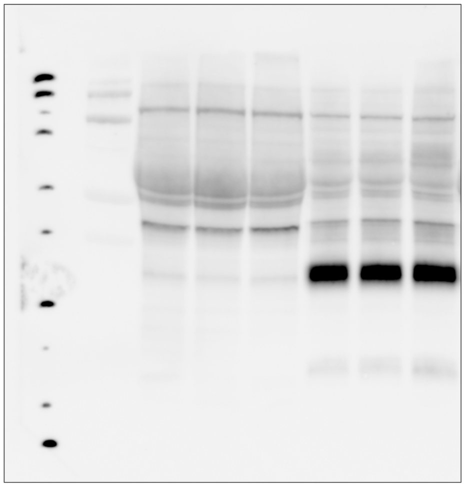

anti-HA

Lysates

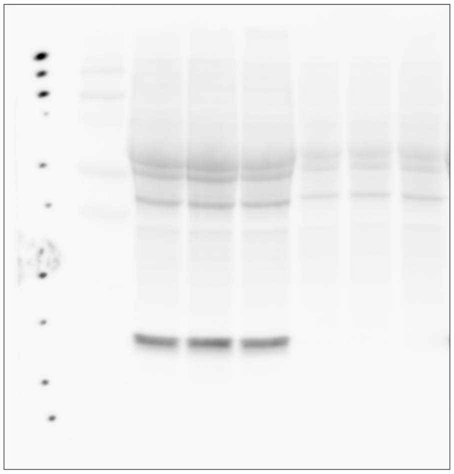

anti-FLAG

Uncropped western blots for Figure 3K

IP-FLAG

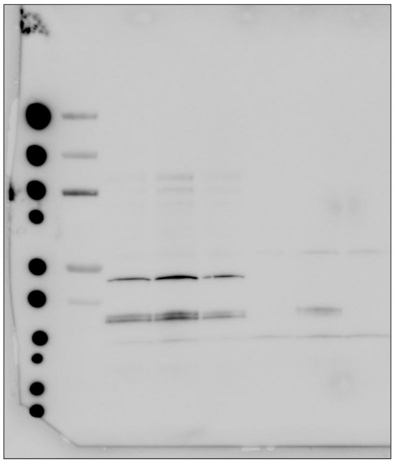

anti-HA

IP-FLAG

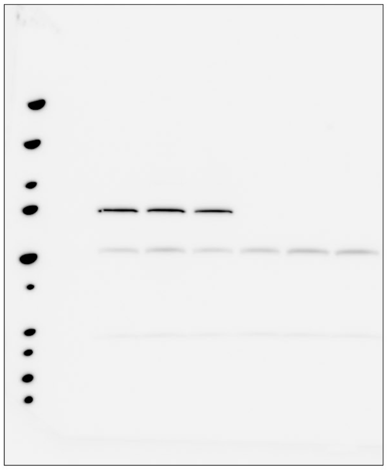

anti-FLAG

Lysates

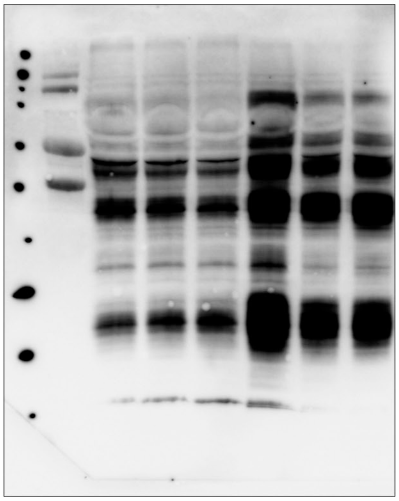

anti-HA

Lysates

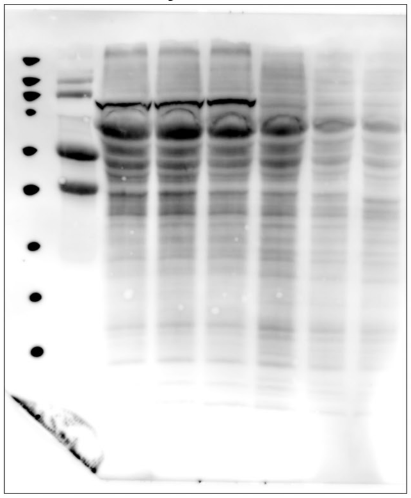

anti-FLAG

Uncropped western blots for Figure 3L

IP-FLAG

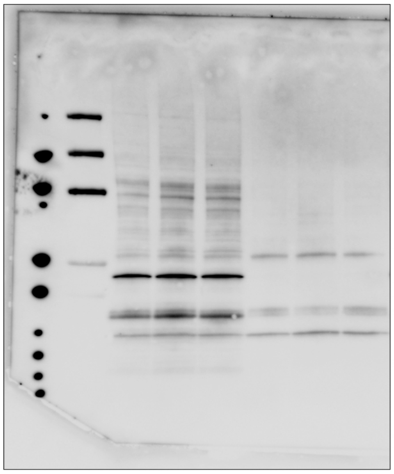

anti-HA

IP-FLAG

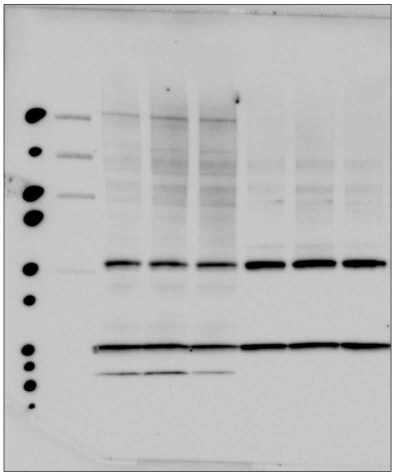

anti-FLAG

Lysates

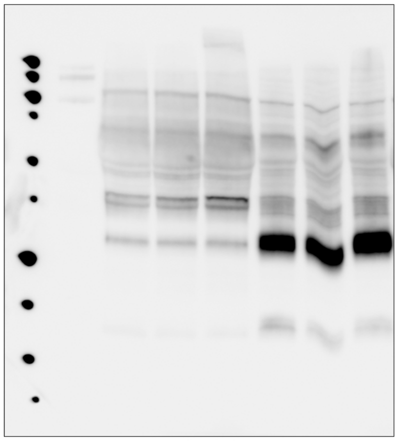

anti-HA

Lysates

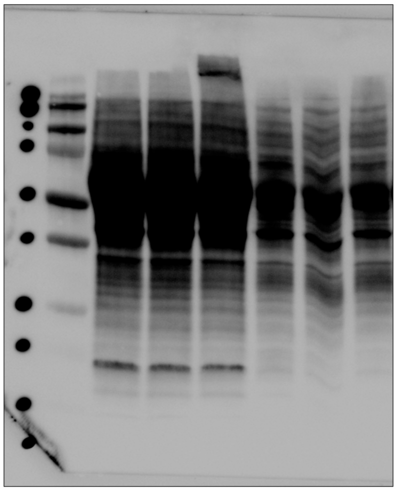

anti-FLAG

Uncropped western blots for Figure 3M

IP-FLAG

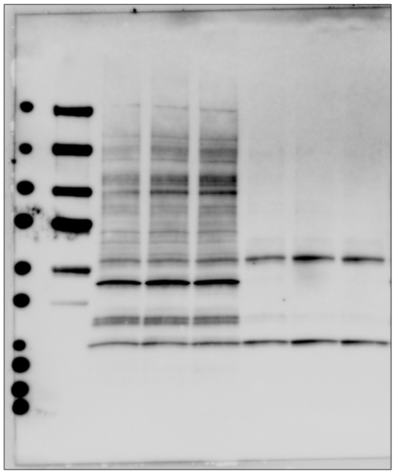

anti-HA

IP-FLAG

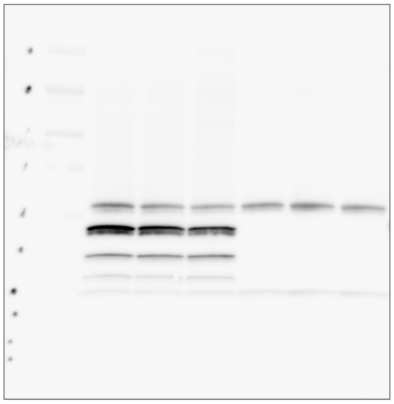

anti-FLAG

Lysates

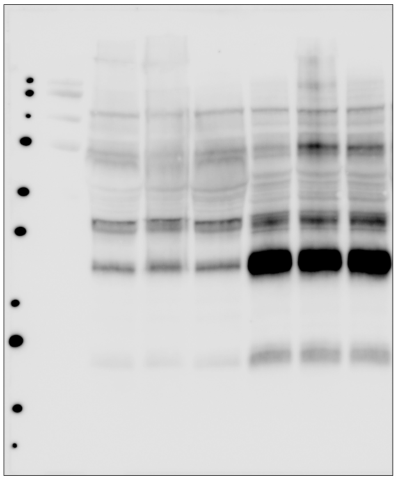

anti-HA

Lysates

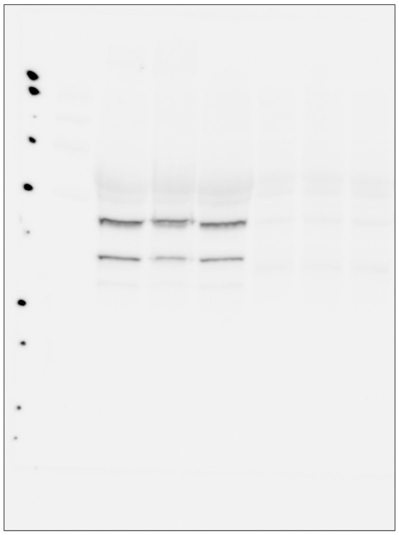

anti-FLAG

Uncropped western blots for Figure 3N

IP-FLAG

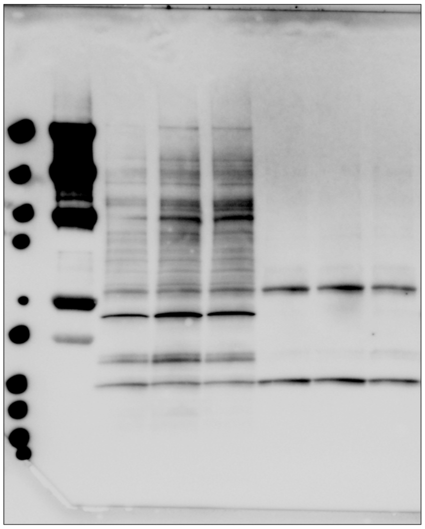

anti-HA

IP-FLAG

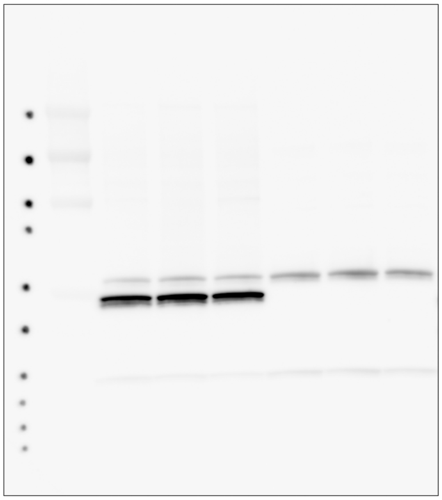

anti-FLAG

Lysates

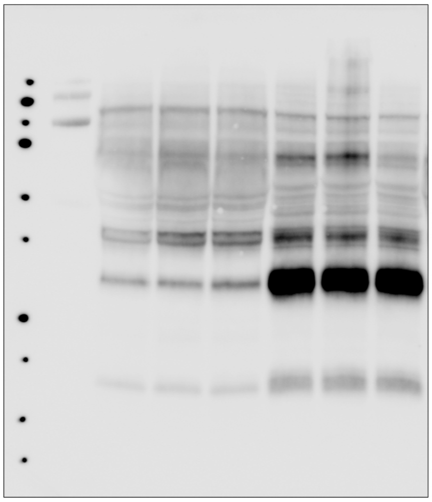

anti-HA

Lysates

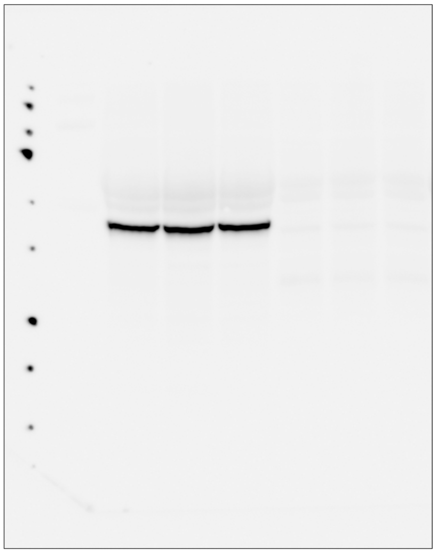

anti-FLAG

Uncropped western blots for Figure 3O

IP-FLAG

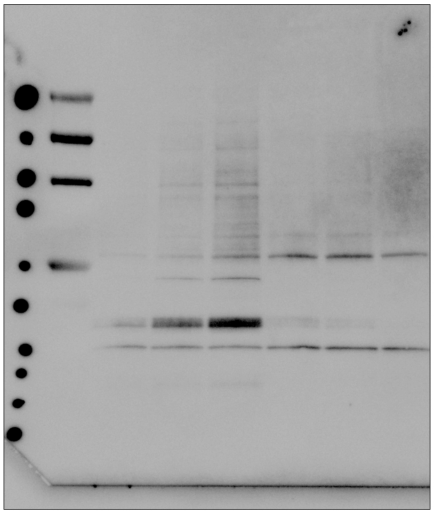

anti-HA

IP-FLAG

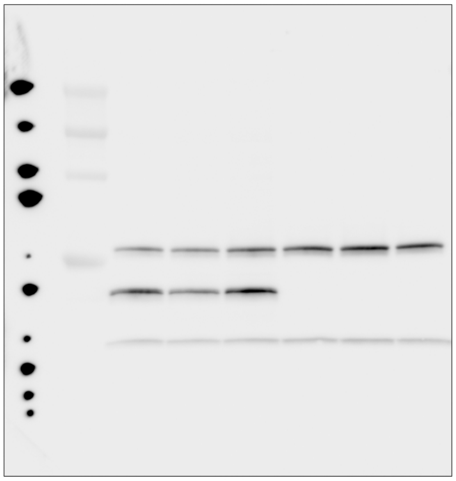

anti-FLAG

Lysates

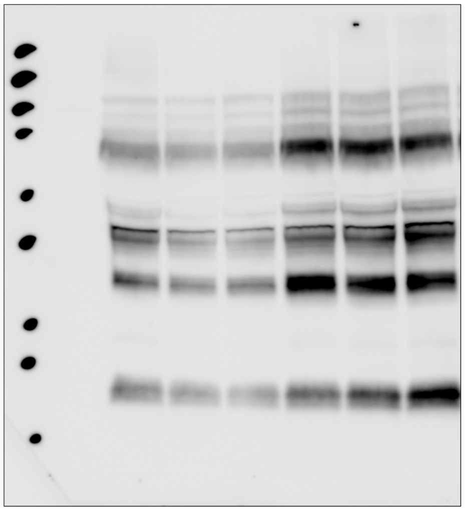

anti-HA

Lysates

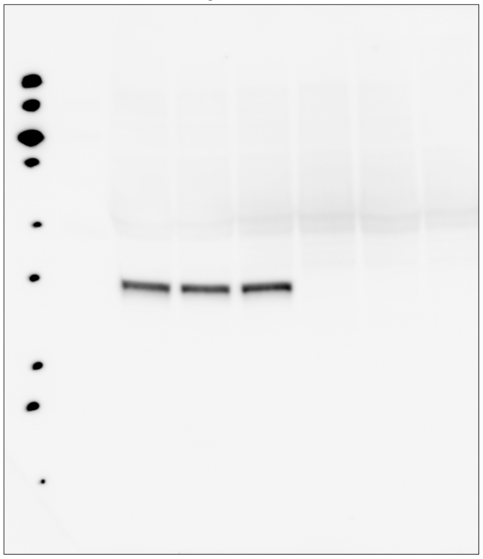

anti-FLAG
